# Operation of MXene-Derived Zinc-Preintercalated Bilayered Vanadium Oxide Cathode in Aqueous Zn-Ion Batteries

**DOI:** 10.1021/acsaem.5c01721

**Published:** 2025-08-27

**Authors:** Timofey Averianov, Kyle Matthews, Xinle Zhang, Huyen T. K. Nguyen, Yuan Zhang, Yury Gogotsi, Ekaterina Pomerantseva

**Affiliations:** † Materials Electrochemistry Group, Department of Materials Science and Engineering, 6527 Drexel University, Philadelphia, Pennsylvania 19104, United States; ‡ A.J. Drexel Nanomaterials Institute Department of Materials Science and Engineering, Drexel University, Philadelphia, Pennsylvania 19104, United States

**Keywords:** chemical preintercalation, vanadium oxide, MXene-derived oxides, aqueous Zn-ion batteries, charge storage mechanism

## Abstract

Layered hydrated vanadium oxides, particularly those with bilayered structures, show remarkable electrochemical performance as cathodes for aqueous Zn-ion batteries (AZIBs). However, their wide-scale adoption is hindered by limited understanding of their charge storage mechanisms in different Zn-containing electrolytes. Here, we demonstrate the first synthesis of a MXene-derived Zn-preintercalated bilayered vanadium oxide (MD-ZVO) with a nanoflower-like morphology comprised of two-dimensional (2D) nanosheets, achieved via a two-step dissolution–recrystallization process. The strategic Zn^2+^ preintercalation establishes well-defined ion diffusion pathways, while the nanoflower-like assembly of 2D nanosheets enhances structural integrity, together contributing to improved electrochemical performance over other layered vanadium oxides. A systematic evaluation of four electrolytes (2 M ZnSO_4_, 2.6 M Zn­(OTf)_2_, 2 M ZnCl_2_, and 30 m ZnCl_2_) showed that MD-ZVO electrodes delivered high reversible capacities (450 and 315 mAh g^–1^ at 0.1 A g^–1^), excellent rate capability (223 mAh g^–1^ for both electrolytes at 1.0 A g^–1^), and good electrochemical stability (84% and 48% over 1000 cycles at 1.0 A g^–1^) in saturated 2.6 M Zn­(OTf)_2_ and highly concentrated 30 m ZnCl_2_, respectively. The material’s superior electrochemical stability in concentrated electrolytes is attributed to suppressed vanadium oxide dissolution during cycling. *In situ* and *ex situ* XRD analyses of MD-ZVO electrodes reveal larger contribution of Zn^2+^-associated species for charge storage in cells containing 2.6 M Zn­(OTf)_2_ and proton dominant charge transfer in cells containing 30 m ZnCl_2_. Additionally, the combination of *in situ* and *ex situ* characterization demonstrates the reversible formation of Zn_*x*_OTf_*y*_(OH)_2*x*–*y*_·*n*H_2_O in cells using 2.6 M Zn­(OTf)_2_ and Zn_5_(OH)_8_Cl_2_·H_2_O in cells using 30 m ZnCl_2_ on the MD-ZVO electrode surface over extended cycling. This work highlights the superior performance of nanoflower MD-ZVO for cathodes in aqueous Zn-ion batteries, which benefits from the proper selection of highly concentrated electrolytes that enable better utilization of the cathode material.

## Graphical abstract

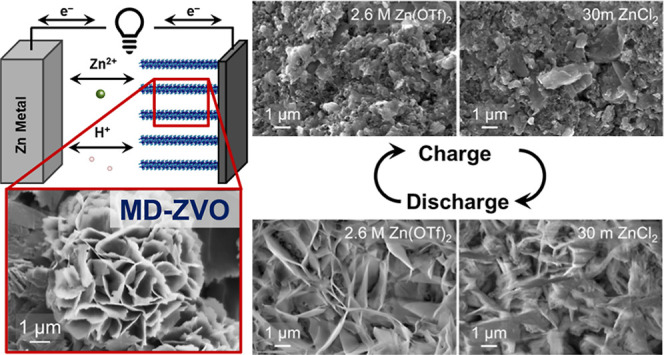

## 1 Introduction

High costs and growing concerns over safety, material scarcity and environmental sustainability increasingly challenge the demand for efficient energy storage systems. While nonaqueous lithium-ion batteries (LIBs) remain dominant in battery energy storage systems,^1^ their sensitivity to fires and reliance on costly raw materials present limitations for practical applications, especially at larger scales. In response, aqueous zinc-ion batteries (AZIBs) have emerged as an attractive alternative. They offer comparable energy densities, improved safety, lower costs, and a more robust supply chain.^2–4^ The aqueous electrolyte chemistries dictate many of the considerations for AZIB design. The water-based electrolytes enable the direct use of a pure zinc–metal anode, which is safer than lithium–metal and exhibits a high theoretical capacity (820 mA h g^–1^) and a low redox potential (−0.76 V vs SHE). This makes it suitable for practical applications without requiring complex host structures.

As in many electrochemical energy storage systems, one of the main challenges for next-generation AZIBs is the development of high-performance cathode materials. Manganese oxides have been widely explored as one of the first cathode materials in both primary^5^ and secondary AZIBs,^6,7^ but they suffer from dissolution in aqueous electrolytes, limiting their long-term stability. Furthermore, the bivalency of Zn^2+^ ions leads to a large solvation shell which can hinder facile intercalation into conventional cathode hosts.^8^ Vanadium oxides have shown promise as cathode materials for Zn-ion storage due to their ability to reversibly shift between multiple oxidation states of vanadium, from V^5+^ to V^3+^. Compared to other vanadium-based compounds, such as vanadates and vanadium phosphates, layered vanadium oxides offer a high degree of structural tunability and favorable ion diffusion kinetics due to their open layered frameworks.^9^ However, these oxides also exhibit limitations such as moderate intrinsic conductivity and structural instability in aqueous environments, especially under deep cycling. Alternative vanadium-based compounds like vanadium phosphates can offer greater structural stability and higher working potentials, yet suffer from sluggish kinetics and lower capacities.^10^ Layered hydrated vanadium oxides (LHVOs) have been extensively studied as AZIB cathodes,^11–13^ with the polymorphs known as chemically preintercalated bilayered vanadium oxides, denoted as δ-M_*x*_V_2_O_5_·*n*H_2_O (MVO, M = Li, Na, K, Mg, Ca, Zn, CTA, BTA, etc.), due to the reversible change in oxidation state of vanadium between +5 and +3, showing superior charge storage properties.^14–16^ While Zn^2+^ intercalation was previously demonstrated for V_2_O_5_ in nonaqueous systems,^17–19^ the first bilayered vanadium oxide cathode for AZIBs was a Zn_0.25_V_2_O_5_·*n*H_2_O produced by Kundu et al. This cathode demonstrated excellent cycling stability at high rates, exhibiting 250 mA h g^–1^ over 200 cycles at a current rate of 4C.^20^ Other MVOs, such as Mg_*x*_V_2_O_5_·*n*H_2_O,^21^ Li_*x*_V_2_O_5_·*n*H_2_O,^22^ Al_*x*_V_2_O_5_·*n*H_2_O,^23^ and K_*x*_(NH_4_)_*y*_V_2_O_5_·*n*H_2_O^24^ among others,^8,11,12^ further demonstrated excellent performance as cathodes in AZIBs.

One option for tuning the synthesis of MVOs is the choice of preintercalated interlayer ions, M. In AZIBs, using Zn^2+^ as the preintercalated species can enhance capacities by creating insertion sites in the interlayer region that are thermodynamically favorable for diffusion of electrochemically cycled Zn^2+^ ions.^25^ For instance, Pang et al. demonstrated that Zn-preintercalated bilayered vanadium oxide (ZVO) electrodes outperformed Na-, K-, and Ca-preintercalated variants in AZIBs.^26^ The choice of preintercalated interlayer ions can also be paired with the selection of vanadium precursors. Using layered transition metal carbides, or MXenes,^27,28^ as precursors for the synthesis of MVOs has emerged as a promising approach.^29–31^ MXenes are effective materials for conversion into oxides due to their nanoscale morphology and chemical reactivity in various solvents,^32–34^ yielding oxide electrode materials with excellent electrochemical performance.^35,36^ Compared to conventional precursors such as orthorhombic V_2_O_5_, V_2_CT_*x*_ MXene-derived MVOs showed enhanced stability in LIBs, owing to the morphological stabilization enabled by flower-like nanoparticles composed of two-dimensional (2D) nanopetals.^29^ Recent studies have demonstrated the viability of MXene-derived MVOs in AZIBs. Partial oxidation of V_2_CT_*x*_ resulted in the formation of a VO_*x*_/V_2_CT_*x*_ heterostructure with improved capacities.^37–39^ In the presence of Mn, this structure is further stabilized, delivering capacities up to 289.6 mAh g^–1^ at 10 A g^–1^.^40^ Complete conversion of V_4_C_3_T_*x*_ in the presence of Mn^2+^ and Zn^2+^ results in MVOs but with a one-dimensional (1D) nanobelt morphology that exhibited slightly lower capacities of 218 and 188 mA h g^–1^ at 10 A g^–1^, respectively.^41,42^ Notably, none of these reports mention a nanoflower morphology, which may explain the limited electrochemical stability of these materials as cathodes in AZIBs.

In addition to structural considerations, electrolyte chemistry plays a crucial role in MVO stability in AZIBs. Due to the high polarity of water molecules, they can cointercalate with Zn^2+^ ions and destabilize the MVO lattice by attacking weakly coordinated V–O bonds, leading to MVO dissolution in electrolyte during battery operation.^43,44^ While early AZIB studies used ZnSO_4_-based electrolytes, the Zn­(OTf)_2_ (zinc triflate, OTf = CF_3_SO_3_) electrolyte has become the standard for MVOs and other AZIBs.^12,45^ The large CF_3_SO_3_^–^ anions coordinate around Zn^2+^ cations, shielding them from interactions with water molecules, which not only improves Zn^2+^ transport during cycling, but also prevents unwanted water cointercalation that initiates cathode dissolution.^45^ Highly concentrated electrolytes can further suppress water activity; however, the maximum concentration of Zn­(OTf)_2_ in water is typically limited to a 3 M solution. In pursuit of high-concentration electrolytes for AZIBs, a 30 m ZnCl_2_ “water-in-salt” (WIS) electrolyte has emerged as a cost-effective alternative,^45,46^ reducing water content, limiting byproduct formation, and enhancing capacity retention.^47^ Despite some positive outcomes achieved through minimizing water content, its presence in electrolytes remains essential for ion transport, making electrolyte selection key for long-term stability.

Another important factor related to the operation of the cathode materials in AZIBs is their charge storage mechanism. Three main processes have been identified to govern the cycling of MVOs: the intercalation of Zn^2+^ ions, the cointercalation of protons, and the formation of byproducts. The expanded interlayer region of MVOs’ structure enables the reversible intercalation of the solvated^48^ and unsolvated^20^ Zn^2+^ ions, aided by water’s charge-shielding effect that improves ion diffusion. Chemical preintercalation of Zn^2+^ ions also reduces lattice stress during AZIB cycling by defining favorable ion pathways.^25^ As the primary Zn-containing electrolytes, like ZnSO_4_ and Zn­(OTf)_2_, are weakly acidic, proton cointercalation can occur alongside Zn^2+^ insertion during cycling. The balance between Zn^2+^ and H^+^ participation can be tuned by adjusting the electrolyte pH; more acidic electrolytes favor protons for charge transfer, while raising the pH suppresses proton intercalation.^49^ Additionally, water splitting (H_2_O → H^+^ + OH^–^), accompanied by proton intercalation into the cathode structure, can buffer pH and accumulate OH^–^ ions, which react with dissolved zinc salts in electrolyte to form layered double hydroxide byproducts, such as Zn_4_SO_4_(OH)_6_·5H_2_O (ZHS) and Zn_12_(OTF)_9_(OH)_15_·*n*H_2_O (ZBS).^11,50,51^ The poor reversibility of the byproduct formation over extended cycling was reported as a possible cause for the capacity decay exhibited by AZIBs. While byproduct formation has been shown in dilute ZnCl_2_ electrolytes,^50^ the mechanisms in concentrated 30 m ZnCl_2_ WIS electrolytes remains poorly understood. Despite significant progress, there is still insufficient knowledge related to the charge storage mechanism of ZVO in various aqueous AZIB electrolytes, warranting further systematic study.

In this work, we for the first time report the synthesis of Zn-preintercalated MXene-derived bilayered vanadium oxide (MD-ZVO) with a morphology composed of 2D nanoflakes assembled into nanoflower-like particles. MD-ZVO was prepared using a V_2_CT_*x*_ MXene precursor through a two-step process involving MXene dissolution with hydrogen peroxide in the presence of Zn^2+^ ions, followed by hydrothermal treatment (Scheme 1). To establish electrochemical charge storage properties of MD-ZVO cathodes in aqueous Zn-ion cells, it was cycling against a Zn metal anode with four electrolytes: 2 M ZnSO_4_, 2.6 M Zn­(OTf)_2_, 2 M and 30 m ZnCl_2_ in water. The mechanism of the MD-ZVO charge storage was analyzed *via* a combination of *in situ* and *ex situ* characterization methods, allowing to monitor intercalation/deintercalation and byproduct formation/decomposition processes.

**Scheme 1:**
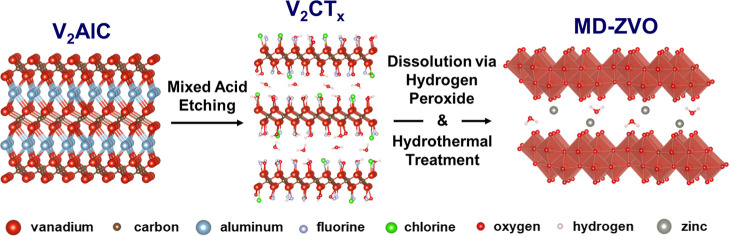
Synthesis Schematic of the Three-step V_2_AlC to V_2_CT_*x*_ to δ-Zn_0.19_V_2_O_5_·0.57H_2_O Transformation Process. The V_2_AlC MAX Phase is Chemically Etched Using a Mixed Acid Etchant Composed of Water, Hydrochloric Acid, and Hydrofluoric Acid, Followed by a Washing Step, Producing the Multilayer V_2_CT_*x*_ MXene. The V_2_CT_*x*_ MXene is Dispersed in a 0.5 M ZnCl_2_ Solution and Dissolved Using Hydrogen Peroxide. After Some Stirring, the Solution is Hydrothermally Treated to Recrystallize the Dissolved MXene and ZnCl_2_ Into MD-ZVO

## 2 Experimental Methods

### 2.1 Synthesis of V_2_AlC MAX Phase and V_2_CT_*x*_ MXene Precursors

The synthesis procedure for the V_2_AlC MAX phase and V_2_CT_*x*_ MXene were adapted from a previous report.^29^ For V_2_AlC MAX phase, vanadium metal powder (99.5%, 325 mesh, Alfa Aesar), aluminum metal powder (99.5%, 325 mesh, Alfa Aesar), and graphite (99%, 325 mesh, Alfa Aesar) were mixed together in a 2:1.1:0.9 atomic ratio and ball-milled with 10 mm zirconia balls (2:1 ball/powder ratio) in plastic jars at 50 rpm for 18 h. The milled mixture was transferred to alumina crucibles that were placed in a tube furnace (Carbolite Gero). Ar gas (200 cm^–3^) was flown through the furnace for 1 h before heating and continuously flown during the annealing process. The temperature was raised to 1550 °C at 3 °C min^–1^, held at 1550 °C for 2 h, and cooled to room temperature at 3 °C min^–1^. The resulting MAX phase slab was then ground down and sieved to a particle size range of 20–32 μm. For V_2_CT_*x*_ MXene, a mixed acid etchant consisting of deionized (DI) water, hydrochloric acid (HCl, 36.5–38%, Fisher Chemical), and hydrofluoric acid (HF, 48.5–51%, Acros Organics) was prepared in a 6:12:12 volume ratio in a high-density polyethylene (HDPE) bottle with a Teflon-coated magnetic stir bar on a hot plate. For each 30 mL of this etchant, 1 g of 20–32 μm sieved V_2_AlC was slowly added to the solution over 5 min. The powder was stirred at 300 rpm at 35 °C for 120 h. After etching, the mixture was split between two 150 mL polypropylene centrifugation tubes, filled with deionized water, and redispersed for homogeneity. These tubes were centrifuged at 3500 rpm for 5 min, and the supernatant was poured off. The remaining MXene sediment was redispersed with water, and the washing process was repeated until the pH of the supernatant registered ≥6.

### 2.2 Synthesis of MXene-Derived Zn_*x*_V_2_O_5_·*n*H_2_O

The synthesis procedure of MXene-derived δ-Zn_*x*_V_2_O_5_·*n*H_2_O (MD-ZVO) was adapted from a previous report.^29^ First, 30 mL of 0.5 M ZnCl_2_ (98+%, anhydrous, Thermo Chemical) was prepared in a flask at room temperature. At this low concentration, a white precipitate may form due to impurities in the stock ZnCl_2_; however, it redissolves later in the synthesis process and does not appear in the final product. Then, 300 mg of multilayer V_2_CT_*x*_ powder was added to the solution, followed by the addition of 2 mL of 30 wt % hydrogen peroxide (Fisher Scientific). This mixture was stirred at room temperature at 300 rpm for 2 h. The resulting dark green solution, characteristic of the H_2_O_2_-induced transformation of V_2_CT_*x*_ into oxides,^29–31^ was allowed to settle briefly to precipitate any unreacted MAX phase. The supernatant was then transferred into two Teflon-lined acid digestion vessels (model 4749, 23 mL, Parr Instrument Company), with 15 mL of solution in each. The vessels were placed in a gravity oven for hydrothermal treatment at 140 °C for 24 h. Afterward, the precipitate that formed in the digestion vessels was collected, dispersed in 100 mL of DI water, vacuum filtered, and washed with an additional 200 mL of DI water. The washed precipitate was dried at 105 °C in air to obtain the final product.

### 2.3 Materials Characterization

The morphology and chemical composition of the MD-ZVO was probed using a ThermoFisher Apreo 2 S scanning electron microscope equipped with a Trinity Detection in-lens and in-column electron detector system and a ThermoFisher UltraDry energy-dispersive X-ray spectroscopy (EDS) detector. Samples were mounted on Al stubs with carbon tape and sputter-coated with a ∼5 nm thick layer of Pt/Pd to reduce surface charging and improve image quality. EDS spectra were collected using a 10 kV acceleration voltage at a magnification of 10000× to evaluate the chemical composition of the synthesized material. Interlayer zinc content was also determined using an AA-7000 atomic absorption spectrometer (AAS, Shimadzu, Japan). Calibration curves were generated using zinc standard solutions at concentrations of 0.1, 0.2, 0.5, 1.0, and 1.5 μg mL^–1^ and vanadium standard solutions at 5, 10, 15, and 20 μg mL^–1^. Both sets of standard solutions were prepared by diluting 1000 μg mL^–1^ stock solutions (Inorganic Ventures, USA) with analytical-grade water. For AAS sample preparation, approximately 10 mg of ground MD-ZVO powder was dispersed in a beaker containing deionized water, followed by the addition of one drop of 30 wt % H_2_O_2_ (Alfa Aesar, USA) to aid dissolution. The mixture was sonicated for 10 min and subsequently graduated in a 100 mL volumetric flask. The final AAS sample was prepared by further diluting 20 mL of the prepared solution to 100 mL with deionized water. Zinc absorptions were measured using acetylene-air fuel, and vanadium absorptions were measured using an acetylene-N_2_O fuel with flame optimization. Each cathode lamp was warmed up for 5 min before AAS measurements. The phase composition of MD-ZVO was determined using a Rigaku Miniflex benchtop X-ray diffractometer with Cu Kα (λ = 1.54 Å) radiation. Patterns were collected using a step size of 0.02° and a step acquisition time of 0.5 s. Water content was estimated using a TGA Q50 thermogravimetric analyzer. Thermal weight loss was analyzed from room temperature to 1000 °C at a heating rate of 10 °C min^–1^ under an ambient air environment. Oxidation state information was obtained using a Physical Electronics VersaProbe 5000 X-ray photoelectron spectrometer (XPS). The high-resolution V 2p, O 1s, and Zn 2p spectra were taken at a pass energy of 27.5 eV with a step size of 0.05 eV. Peak fitting and data analysis were carried out using CasaXPS software. A Shirley background was used for spectra analysis. Spectra were calibrated to the oxygen peak at 530 eV. Additional compositional and structural information was collected using a Bruker Invenio-R mid-IR spectrometer and a Renishaw inVia Raman spectrometer (Gloucestershire, UK). Mid-IR spectra were obtained over the range of 400–4000 cm^–1^. Raman spectra were acquired using a 633 nm He–Ne laser with a 1200 line/mm grating and a 20× objective (NA = 0.4). The excitation intensity was 4.06 nW at 50% laser power and an acquisition time of 10 s.

### 2.4 Electrode and Cell Fabrication

MD-ZVO electrodes were fabricated using a slurry casting method. The slurry was comprised of an 80:15:5 mass ratio of the MD-ZVO powder, acetylene carbon black (Alfa Aesar), and polytetrafluoroethylene (PTFE, 60 wt % dispersion in water, Sigma-Aldrich). The oxide and carbon black powders were mixed and ground together in a mortar and pestle for 20 min and mixed in a Flacktek DAC 150.1 FV–K Speedmixer at 3000 rpm for 1 min to homogenize the mixture. PTFE dispersion was added to the mixed powder and mixed again in the Flacktek mixer at 3000 rpm for 1 min. Water was added dropwise to the mixture and mixed once again, repeating until the slurry exhibited the desired viscosity. The final slurry was spread in a line on a prepared slice of carbon paper (TGP-H-60, Toray, Thermo Scientific) and uniformly cast along its length using a doctor blade set to 150 μm. The cast electrode was dried at room temperature in a fume hood overnight, followed by drying at 60 °C in ambient air for at least 12 h. Electrode discs were then punched out using a 3 mm diameter punch. Aqueous Zn-ion half cells were assembled using Swagelok perfluoroalkoxy (PFA) T cells as the cell body. The MD-ZVO electrode discs served as the working electrodes, while 100 μm Zn foil (Fisher Chemicals) punched into 5 mm diameter discs and rolled to a thickness of ∼50 μm acted as the counter electrodes. Zn metal electrodes required no additional cleaning or processing for use in AZIBs. 3 mm diameter glassy carbon electrodes (CH instruments) acted as the current collectors. Glass microfiber filters (Whatman) were used as separators. Four aqueous electrolytes were investigated in this study: 2 M ZnSO_4_ (Thermo Chemicals), 2 M ZnCl_2_, 30 m ZnCl_2_, and 2.6 M Zn­(OTf)_2_ (OTf = CF_3_SO_3_, Thermo Chemicals). Despite numerous reports of 3 M Zn­(OTf)_2_ electrolytes used for AZIBs,^8,52^ the maximum solubility that was achieved in this work was 2.6 M.

### 2.5 Electrochemical Characterization

All potentials in this work are reported vs Zn/Zn^2+^. The aqueous Zn-ion cells were cycled in a potential window of 0.2–1.6 V. Cyclic voltammetry (CV) profiles and electrochemical impedance spectroscopy (EIS) spectra were collected using a BioLogic VMP3 potentiostat. Voltammograms were obtained using scan rates of 0.1, 0.2, 0.4, 0.6, 0.8, and 1.0 mV s^–1^. Extended life cycling and rate capability testing of the aqueous Zn-ion cells was performed on an Arbin Battery Tester. For the extended life galvanostatic discharge/charge (GDC) testing, cells were cycled at current densities of 0.1 and 1.0 A g^–1^ for 350 and 1000 cycles, respectively. For rate capability testing, the cells were cycled at current densities of 0.1, 0.2, 0.5, 1.0, 2.0, 5.0, 10.0, 5.0, 2.0, 1.0, 0.5, 0.2, and 0.1 A g^–1^ for 10 discharge/charge cycles at each current density. EIS was performed by collecting spectra at various points during GDC cycling at a current density of 1.0 A g^–1^. A spectrum was collected at the open circuit voltage (OCV), and then at the end of discharge (0.2 V, 0% state of charge or SOC) and end of charge (1.6 V, 100% SOC) for the first, 10th, and 100th GDC cycles.

### 2.6 *In Situ* and *Ex Situ* Characterization

In situ XRD measurements were performed on a Rigaku SmartLab diffractometer using a custom-built aqueous Zn-ion cell connected to a Pine Instruments WaveNow Potentiostat/Galvanostat portable system. The design of the cell and its implementation in the diffractometer are shown in Figure S1 in Supporting Information. The cell elements were placed into a cell body made of 3M VHB (Very High Bond) 4910 clear tape. The 2.0 cm × 1.0 cm Zn metal anode, with a 2.0 cm × 0.5 cm Zn metal leg serving as the negative terminal connection, was covered by three pieces of 1.0 cm × 0.635 cm glass fiber separator (Whatman 934-AH) that filled in the central cavity of the VHB tape cell body to match the tape thickness as well as provide a solid medium for electrolyte containment. The cavity was then covered with a 1.25 cm × 1 cm piece of Celgard 3501 hydrophilic polypropylene membrane compatible with aqueous electrolytes. A piece of 130 μm thick graphite foil was placed down on the exposed VHB tape body to act as the positive terminal connection to the working electrode, after which the MD-ZVO electrode cast on carbon paper was placed over the Celgard membrane and overlapping the graphite foil. This entire stack was enclosed in a piece of Kapton polyimide film, allowing X-ray penetration for structural analysis. 2.6 M Zn­(OTf)_2_ and 30 m ZnCl_2_ were used as electrolytes for these measurements, which were injected into the cell cavity with a fine-tip needle and syringe. A reasonable time was allowed for the electrolyte to soak the glass fiber membranes, establishing a connection between the cell components. Once an adequate open-circuit voltage (>1.3 V) was achieved, operation commenced immediately. The cell was cycled between 0.2 and 1.6 V at a current density of 0.2 A g^–1^ while diffraction patterns were obtained simultaneously from 3 to 25° 2θ at a step size of 0.02°, a step acquisition time of 0.6 s, and a scan speed of 1.0 °/min.

*Ex situ* XRD, SEM, and XPS measurements were performed on cathodes collected at 0% and 100% SOC on the first and 100th GDC cycles and compared to the relevant characterization of the pristine electrodes (before cycling). Prior to analysis, cycled electrodes were gently rinsed by dipping in DI water under mild agitation to remove residual electrolyte salts while preserving any deposited byproducts, and then dried at 60 °C in ambient air overnight for 12 h.

## 3 Results and Discussion

The successful synthesis of the V_2_AlC MAX phase and V_2_CT_*x*_ MXene multilayer nanoflakes was confirmed by SEM imaging and XRD analysis. The SEM image of the MAX phase shows bulky particles comprised of tightly stacked large two-dimensional (2D) crystals (Figure S2a in Supporting Information), while the SEM image of MXene (Figure S2b in Supporting Information) presents the separation of those 2D crystals leading to thinner 2D agglomerates indicating the removal of the Al atoms and the formation of multilayer MXene nanoflakes.^53,54^ The XRD patterns of the MAX phase and MXene (Figure S2c in Supporting Information) demonstrate the expansion of the layered structure with the emergence of the (002) peak at 9.18° 2θ, characteristic of MXene formation in the process of MAX phase etching. The morphology of the oxide derived from the as-obtained V_2_CT_*x*_ MXene is revealed in the SEM images in Figure 1a. The nanoflower particles, composed of centrally nucleated 2D nanosheets of MXene-derived Zn-preintercalated bilayered vanadium oxide (MD-ZVO), exhibit a morphology consistent with other V_2_CT_*x*_-derived MVOs.^29,31^ This morphology can be observed in different batches of MD-ZVO (Figure S3 in Supporting Information), demonstrating the uniformity and reproducibility of the nanoflower morphology for MXene-derived ZVO obtained using the MXene-to-oxide transformation approach. This distinct shape of the particles differs from the morphologies typically observed in similar materials synthesized from non-MXene precursors.^55,56^ To the best of our knowledge, this is the first report of a nanoflower morphology for a Zn-preintercalated bilayered vanadium oxide. Most previously reported ZVOs, including those synthesized from other MXene precursors,^42^ represent one-dimensional (1D) nanoparticles such as nanorods or nanobelts.^20,56^ EDS mapping of the MD-ZVO surface (Figure 1b) shows a strong spatial overlap between the O and the Zn signals, indicating a uniform distribution of the interlayer Zn^2+^ ions.

**Figure 1:**
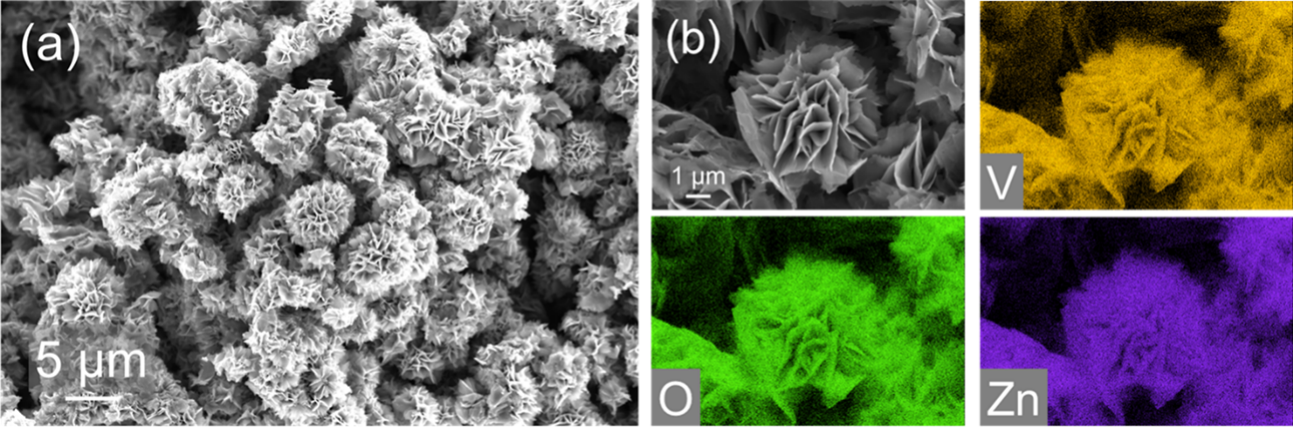
Morphology and composition of V_2_CT_*x*_ MXene-derived Zn-preintercalated bilayered vanadium oxide (MD-ZVO): (a) SEM image of MD-ZVO nanoflowers and (b) SEM image of MD-ZVO with corresponding EDS mapping for V, O, and Zn.

The composition of MD-ZVO was determined based on EDS and atomic absorption spectroscopy (AAS) analysis to establish the Zn/V ratio and TGA analysis to determine the hydration degree. From the analysis of the EDS spectra for MD-ZVO (Figure S4 in Supporting Information), the Zn/V ratio was determined as 0.096, which translates to an *x* value in δ-Zn_*x*_V_2_O_5_·*n*H_2_O of 0.19. The interlayer Zn^2+^ content calculated using AAS measurements (Figure S5 in Supporting Information) produced a Zn/V ratio of 0.086 that translates to an *x* value of 0.17. Even though EDS is primarily a surface technique and AAS is a bulk technique, we observed good agreement between the two measurement methods which has been previously demonstrated for chemically preintercalated bilayered vanadium oxides,^31,57^ and confirm the uniform distribution of Zn^2+^ ions in the interlayer regions of the bilayered vanadium oxide structure. In the TGA weight loss curve for MD-ZVO (Figure 2a), the interlayer water content (*n* in δ-Zn_*x*_V_2_O_5_·*n*H_2_O) corresponds to the weight loss observed in the temperature range of 100–500 °C, similarly to other chemically preintercalated bilayered vanadium oxides.^29,58^ Below these temperatures, the weight loss is related to weakly bound adsorbed or physisorbed water, while above this range, the weight loss likely corresponds to the oxygen evolution due to the degradation of the MD-ZVO structure.^58^ The weight loss of 4.98% corresponds to an *n* value of 0.57, giving an overall chemical composition of δ-Zn_0.19_V_2_O_5_·0.57H_2_O for MD-ZVO. The XRD pattern of MD-ZVO (Figure 2b) was indexed to the Zn_0.25_V_2_O_5_·*n*H_2_O triclinic phase (JCPDS: 86-1238)^20^ with a (001) *d*-spacing of 10.42 Å, which is in fair agreement with the (001) *d*-spacing calculated through Bragg’s law for MD-ZVO of 10.753 Å. The deviation in *d*-spacing value is likely due to the differences in interlayer Zn^2+^ and water content.^31^ The FTIR (Figure 2c) and the Raman (Figure 2d) spectra of MD-ZVO show the vibrational features related to the layered hydrated vanadium oxide (LHVO) structure. In the FTIR spectrum, the LHVO fingerprint region in the range of 400–1000 cm^–1^ contains stretching modes for V–O–V (538 and 766 cm^–1^) and V=O (1005 cm^–1^) bonds, while the features at 1620, 3458, and 3572 cm^–1^ correspond to the O–H bending and stretching modes of the interlayer water.^59^ The Raman spectrum only contains LHVO structure-related fingerprint features as water is Raman inactive, with the skeletal bending mode (153 cm^–1^), the bending (264 and 416 cm^–1^) and stretching (904 and 1017 cm^–1^) modes of the V=O bonds, and the stretching modes of the V–O bonds (512 and 691 cm^–1^).^59^ The presence of these features, as well as the low spectral noise for both spectra, indicates the high crystallinity of MD-ZVO. The positions of the fingerprint region peaks and the relative intensities of the water-related features in the FTIR spectrum indicate a well-hydrated LHVO structure, which is supported by the water content determined through TGA.^59^ The slight emergence of the Raman peak at 904 cm^–1^ also indicates that some of the terminal oxygens are replaced with the oxygen from the water molecule, which can impact the transport of ions during AZIB cycling.^48,59^ Additionally, past the fingerprint region in the Raman spectra (Figure S6 in Supporting Information), a small band was observed at 1559 cm^–1^, which is usually attributed to the presence of carbon in the sample. Based on the EDS analysis (Figure S4 in Supporting Information), only 2.3% of carbon was detected, implying that this residual carbon may be in the form of some adventitious carbon or unreacted MAX phase or MXene that does not appear in the XRD pattern of MD-ZVO.

**Figure 2:**
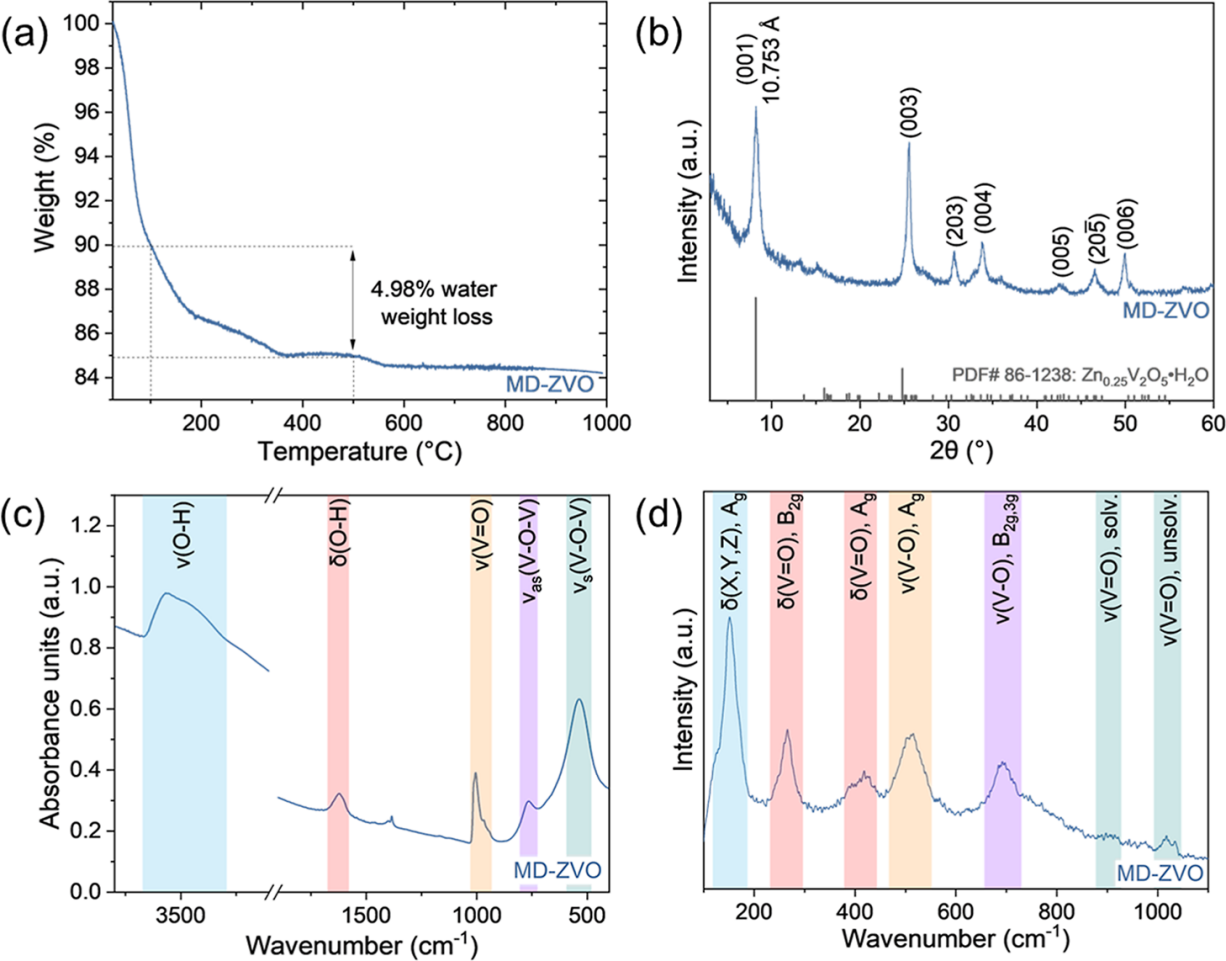
Structural analysis of MD-ZVO: (a) TGA weight loss curve, (b) XRD pattern with associated JCPDS card # 86–1238, (c) FTIR spectrum, and (d) Raman spectrum.

Cyclic voltammetry (CV) profiles of the cells containing MD-ZVO electrodes (Figure 3a–d) depict the electrochemical behavior of the MXene-derived oxide in four different electrolytes: 2 M ZnSO_4_, 2.6 M Zn­(OTf)_2_, 2 M ZnCl_2_, and 30 m ZnCl_2_, all in water. The features for each of the CV profiles can be divided into two groups: one set of reversible peaks appearing at 0.2–0.7 V and a second set of reversible peaks positioned at 0.7–1.6 V. The presence of multiple peaks indicates that the charge storage mechanism is a multistep process, as has been previously identified for MVOs in AZIBs.^55,60^ The CV profile of the cell containing the 2.6 M Zn­(OTf)_2_ electrolyte shows the largest number of identifiable peak pairs among the four electrolytes studied in this work (Table S1 in Supporting Information); eight cathodic/anodic peak pairs were observed, two in the lower half of the voltage range (0.44 V/0.56 and 0.62 V/0.74 V) and six in the upper half of the range (0.79 V/1.00 V, 0.98 V/1.07 V, 1.13 V/1.14 V, 1.18 V/1.21 V, 1.34 V/1.37 V, and 1.36 V/1.44 V). Peaks at similar positions appear in the CV profiles of the cells with the other electrolytes, indicating similarities in the electrochemical processes that occur between the cathode and the electrolyte. The intensities of the peaks also show similarities, most notably for the two peak pairs appearing at lower potentials, which may indicate a common electrochemical process among all four electrolytes. The most unique response among the four electrolytes is shown by the cell containing 30 m ZnCl_2_. In the CV profile of the cell containing 30 m ZnCl_2_ electrolyte, there are practically no peaks appearing above 1.2 V, and a larger rectangular area is observed instead, which could be attributed to a less diffusion-limited charge storage mechanism governed by the concentrated 30 m ZnCl_2_ electrolyte. The peak positions in the CV profiles for the 30 m ZnCl_2_ cell also appear shifted to higher potentials compared to other electrolytes, which is consistent with the increase in activity of Zn^2+^-containing charge-carrying species in accordance with the Nernst equation, as reported for highly concentrated electrolytes in AZIBs.^46,47^ Cells containing the four electrolytes were also galvanostatically cycled at a low current density of 0.1 A g^–1^ to evaluate their relative electrochemical stability. Over 350 cycles, it was revealed that the cells containing 2.6 M Zn­(OTf)_2_ and 30 m ZnCl_2_ maintained the highest specific capacities. While the cells containing 2 M ZnSO_4_ and 2 M ZnCl_2_ demonstrated initial capacities of 337 and 397 mAh g^–1^, respectively, after 350 cycles, these cells delivered only 29.6 (9% capacity retention) and 52.3 mAh g^–1^ (13% capacity retention). The rapid capacity decay was primarily attributed to the dissolution of vanadium in dilute and mildly acidic electrolytes.^11^ In contrast, the cell containing 2.6 M Zn­(OTf)_2_ electrolyte demonstrated a high initial specific capacity of 450 mA h g^–1^ and maintained a capacity of 261 mAh g^–1^ after 350 cycles (capacity retention of 58%). The cell containing 30 m ZnCl_2_ exhibited an initial capacity of 315 mAh g^–1^ and a capacity of 187 mAh g^–1^ after 350 cycles, corresponding to a similar 59% capacity retention. Interestingly, the capacity decay profile is significantly different for the cells containing 2.6 M Zn­(OTf)_2_ and 30 m ZnCl_2_, as the 30 m ZnCl_2_-containing cell exhibited a sharp decrease in capacity within the first 10 cycles followed by slower decay whereas the capacity demonstrated by the cell containing 2.6 M Zn­(OTf)_2_ electrolyte showed a more gradual decay over the 350 cycles. Nevertheless, the higher concentration 2.6 M Zn­(OTf)_2_ and 30 m ZnCl_2_ electrolytes showed improved electrochemical stability over the dilute 2 M ZnSO_4_ and 2 M ZnCl_2_, which could be attributed to the suppressed dissolution of the MD-ZVO active material in highly concentrated or saturated electrolytes.^45,61^ The solvation structure of Zn^2+^ ions in these electrolytes limits the cointercalation of water, which is primarily responsible for vanadium dissolution and structural decay.^11^ Due to the excellent combination of high specific capacity and electrochemical stability, we chose the 2.6 M Zn­(OTf)_2_ and 30 m ZnCl_2_ electrolytes for further study.

**Figure 3:**
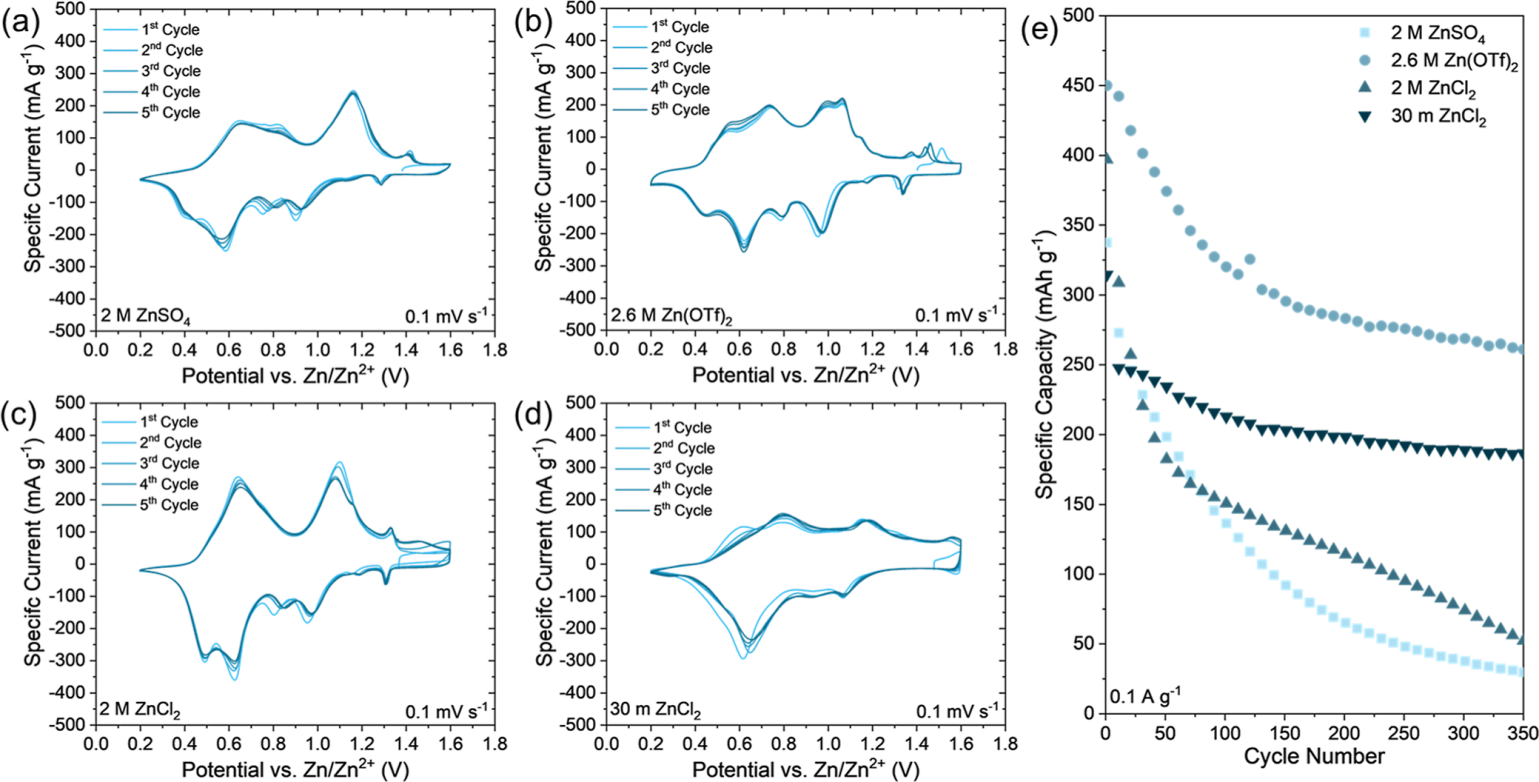
Cycling performance of aqueous Zn-ion cells with MD-ZVO cathode in various electrolytes. (a–d) CV profiles collected at 0.1 mV s^–1^ using (a) 2 M ZnSO_4_, (b) 2.6 M Zn­(OTf)_2_, (c) 2 M ZnCl_2_, and (d) 30 m ZnCl_2_ electrolytes; (e) extended life galvanostatic discharge/charge cycling at 0.1 A g^–1^ (specific capacity for every 10th cycle is plotted).

Scan-rate-dependent CV profiles of the cells containing 2.6 M Zn­(OTf)_2_ and 30 m ZnCl_2_ electrolytes (Figure 4a,b) reveal differences in the rate-dependent electrochemical charge storage mechanism of MD-ZVO nanoflowers. For the cell containing the 2.6 M Zn­(OTf)_2_ electrolyte (Figure 4a), the shape of the CV profiles, exhibiting three well-pronounced peaks, is maintained as the scan rate increases from 0.1 mV s^–1^ to 1.0 mV s^–1^, while some of the more narrow peaks, observed at a scan rate of 0.1 mV s^–1^ appear to merge at higher scan rates in conjunction with the peaks broadening. Additionally, as the sweep rate increases, the peaks shift only slightly, with no significant increase in overpotential (Δ*V* < 0.125 V at 1.0 mV s^–1^). For the cell containing the 30 m ZnCl_2_ electrolyte (Figure 4b), the redox peaks broaden significantly as the scan rate increases from 0.1 mV s^–1^ to 1.0 mV s^–1^, resulting in a larger rectangular area at 1.0 mV s^–1^. Moreover, the broadened peaks appear to shift apart more compared to the peaks in the CV profile of the 2.6 M Zn­(OTf)_2_-containing cell at 1.0 mV s^–1^, exhibiting an increased overpotential (Δ*V* = 0.262 V at 1.0 mV s^–1^) which is attributed to hindered charge transport in this system. Additionally, the evolution of the shape of the CV profiles at increasing scan rates indicates differences in the charge storage mechanism of the MD-ZVO nanoflowers specific to each electrolyte. While both electrolytes use Zn^2+^ ion and proton carrier species for charge transfer, the low pH of concentrated ZnCl_2_ electrolyte (pH < 0) would indicate the higher concentration of protons available for electrochemical interaction as compared to saturated Zn­(OTf)_2_ (3 < pH < 4).^61^ Thus, the 30 m ZnCl_2_ electrolyte relies more on proton intercalation that produces a more nondiffusion-limited charge storage response compared to the 2.6 M Zn­(OTf)_2_ electrolyte which uses both Zn^2+^ and proton species to deliver a more redox-active or diffusion-limited charge storage behavior.^45^ These aspects of the charge storage mechanism in AZIBs closely match our observations in the changes in the CV profile shape at elevated scan rates.

**Figure 4:**
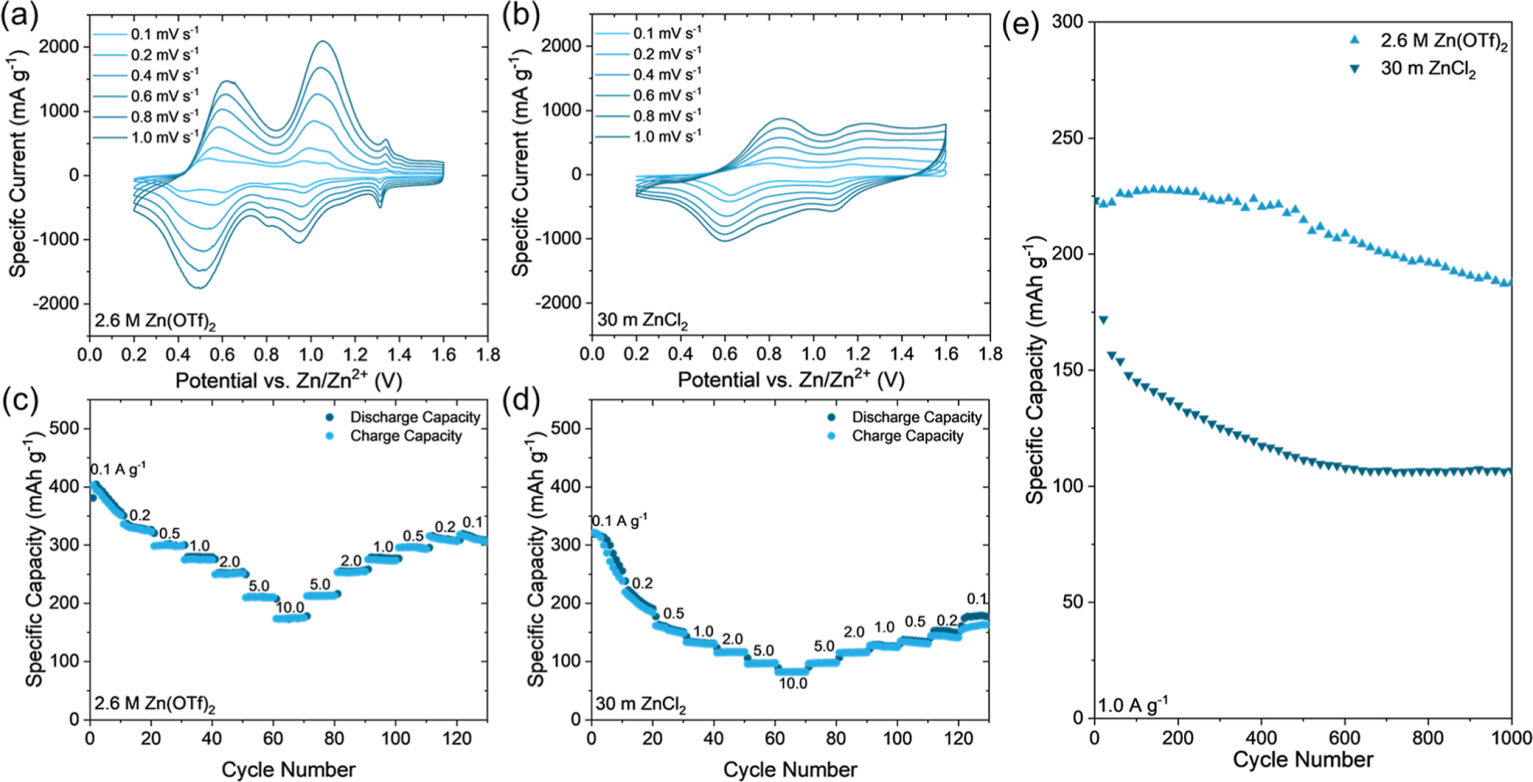
Rate capability and galvanostatic high-rate testing of the aqueous Zn-ion cells with MD-ZVO cathode and selected electrolytes. (a,b) Scan-rate dependent cyclic voltammetry profiles of cells containing (a) 2.6 M Zn­(OTf)_2_ and (b) 30 m ZnCl_2_ electrolytes. (c,d) Rate capability testing of cells containing (c) 2.6 M Zn­(OTf)_2_ and (d) 30 m ZnCl_2_ electrolytes. (e) Extended life galvanostatic discharge/charge cycling performed at a current density of 1.0 A g^–1^ (specific capacity for every 20th cycle is plotted).

The tolerance of the MD-ZVO nanoflowers to high currents in 2.6 M Zn­(OTf)_2_ and 30 m ZnCl_2_ electrolytes, which is important for fast charging, was investigated in rate capability experiments and revealed further disparities between the two electrolytes (Figure 4c,d). The cell containing 2.6 M Zn­(OTf)_2_ (Figure 4c) initially delivered specific capacities of over 400 mAh g^–1^ at 0.1 A g^–1^ while maintaining 174 mAh g^–1^ at 10.0 A g^–1^. By comparison, the cell containing 30 m ZnCl_2_ (Figure 4b) showed a more significant drop in capacity over 322 mAh g^–1^ at 0.1 A g^–1^ to 82 mAh g^–1^ at 10.0 A g^–1^. In 2.6 M Zn­(OTf)_2_, weakly coordinated triflate anions promote more facile Zn^2+^ desolvation and diffusion, which can enhance ion mobility and electrode kinetics, particularly at high current rates.^45^ In contrast, strong Zn–Cl^–^ coordination in 30 m ZnCl_2_ electrolyte can hinder ion mobility, exacerbate concentration polarization and slow down kinetics of the processes at the electrode/electrolyte interface, especially under high-rate conditions, leading to pronounced capacity fade.^61^

To evaluate the extended cycling stability of MD-ZVO under elevated current conditions, we selected a current density of 1.0 A g^–1^ based on the rate capability results, which showed reasonable specific capacities in both electrolytes (Figure 4e). The cell containing the 2.6 M Zn­(OTf)_2_ electrolyte delivered an initial capacity of 223 mAh g^–1^ while maintaining 188 mAh g^–1^ after 1000 cycles, resulting in a capacity retention of 84%. The cell containing the 30 m ZnCl_2_ electrolyte similarly exhibited an initial capacity of 223 mAh g^–1^ but only retained 107 mAh g^–1^ after 1000 cycles, resulting in a 48% capacity retention. The improved capacity retention of the cell containing 2.6 M Zn­(OTf)_2_ over the 30 m ZnCl_2_ electrolyte can again be attributed to the higher ion mobility and charge transport kinetics. Despite the rapid initial capacity decay, the capacities stably maintained by the 30 m ZnCl_2_-containing cell after hundreds of discharge/charge cycles present relatively high values for a current density of 1.0 A g^–1^, and together with the typically lower cost of ZnCl_2_ over Zn­(OTf)_2_, make highly concentrated ZnCl_2_ electrolytes notably promising in applications where cost at scale becomes a limiting factor. In contrast, Zn­(OTf)_2_-based electrolytes are excellent candidates for smaller form factor batteries, where energy and power density are at a premium.

The structural evolution of MD-ZVO electrodes in AZIBs with 2.6 M Zn­(OTf)_2_ and 30 m ZnCl_2_ electrolytes was examined using *in situ* XRD analysis. XRD patterns of MD-ZVO powder, the corresponding electrode placed on the XRD sample holder, and the same electrode assembled into a custom-designed *in situ* XRD cell prior to electrochemical cycling (Figure S7a in Supporting Information) show minimal changes to the MD-ZVO structure with the introduction of the conductive carbon black additive, PTFE binder, and additional cell components. In the XRD patterns of the cell containing 2.6 M Zn­(OTf)_2_ electrolyte (Figure 5a,b), several features were observed over two electrochemical discharge/charge cycles. First, on first discharge, the MD-ZVO (001) peak (highlighted in Figure S7b in Supporting Information) shifted to lower *d*-spacing values, from 10.960 Å to 9.813 Å (Δ*d* = 1.147 Å) and then back to 10.512 Å (Δ*d* = 0.699 Å) on first charge. The second cycle follows a similar trend; a shift to lower *d*-spacing on discharge and a shift to higher *d*-spacing on charge. The contraction and broadening of the MD-ZVO (001) *d*-spacing occurs upon intercalation of cations, as electrostatic attraction between the positively charged intercalating species and the negatively charged MD-ZVO layers leads to a reduction of the interlayer spacing.^20,42,62^ While most reports attribute this contraction and expansion behavior primarily to Zn^2+^ intercalation, the positive singly charged protons may also contribute to this behavior, albeit to a lesser degree. Although the MD-ZVO (001) peak intensity decreases immediately upon the start of cell operation, it remains relatively stable throughout the remainder of the experiment. Several factors may contribute to the observed change in the MD-ZVO (001) peak intensity. One possibility is the gradual wetting of the electrode during the initial discharge, which may lead to electrolyte accumulation on the surface of the electrode and dampening of the X-ray reflection signal. Structural disorder may also arise as water from the electrolyte penetrates the interlayer region and disrupts the stacking of the MD-ZVO layers. Second, a new peak at 6.52° 2θ is observed emerging and disappearing cyclically over the two cycles, which is related to the reversible formation of a Zn_*x*_OTf_*y*_(OH)_2*x*–*y*_·*n*H_2_O (ZTH) layered double hydroxide (LDH), previously identified as a byproduct in AZIBs with vanadium oxide cathodes and Zn­(OTf)_2_ electrolyte.^11,51,63^ Therefore, our *in situ* analysis confirms the previously established mechanism of byproduct formation for such electrochemical systems: on discharge, water from the electrolyte splits into H^+^ and OH^–^ ions, where the protons intercalate into the vanadium oxide structure while the OH^–^ ions react with the Zn^2+^ and OTf^–^ ions and water to form ZTH.^11^ Additionally, the appearance of new peaks at 17.16° and 24.08° 2θ (Figure S8 in Supporting Information) was attributed to the precipitation of Zn­(OTf)_2_ salt from the electrolyte. This suggests that the evolution of the triflate-related peaks during *in situ* XRD cycling may result from water evaporation and/or applied current leading to spontaneous Zn­(OTf)_2_ salt crystallization from the electrolyte. Because the understanding of the solubility behavior of Zn­(OTf)_2_ in aqueous electrolytes remains limited, further investigation is needed to optimize electrolyte formulations for long-term stability of triflate-based AZIBs. Finally, in a separate cell containing 2.6 M Zn­(OTf)_2_ electrolyte (Figure S7d–f in Supporting Information), additional peaks emerged at 12.32° and 20.86° 2θ *ex situ* after the cell was stopped at 1.6 V (100% SOC). These peaks were assigned to Zn_3_V_2_O_7_(OH)_2_·2H_2_O, a zinc pyrovanadate phase that can form in the presence of VO_2_(OH)^−^ ions under mildly acidic conditions.^64^ The hydrolysis of vanadium oxide, which is possible for MD-ZVO electrodes in aqueous electrolytes, leads to the generation of these ions, representing a major mechanism for vanadium oxide electrodes degradation in AZIBs,^11^ and the resulting pyrovanadate phase has demonstrated limited electrochemical storage capabilities.^65^ The formation of Zn_3_V_2_O_7_(OH)_2_·2H_2_O may also lead to the observed capacity decay of MD-ZVO over extended cycling (Figure 3e). Collectively, these observations confirm the occurrence of major processes established for vanadium oxide cathodes in AZIBs: reversible intercalation of Zn^2+^ ions accompanied by cointercalation of protons, precipitation of electrolyte-derived species, and formation of inactive byproduct phases.

**Figure 5:**
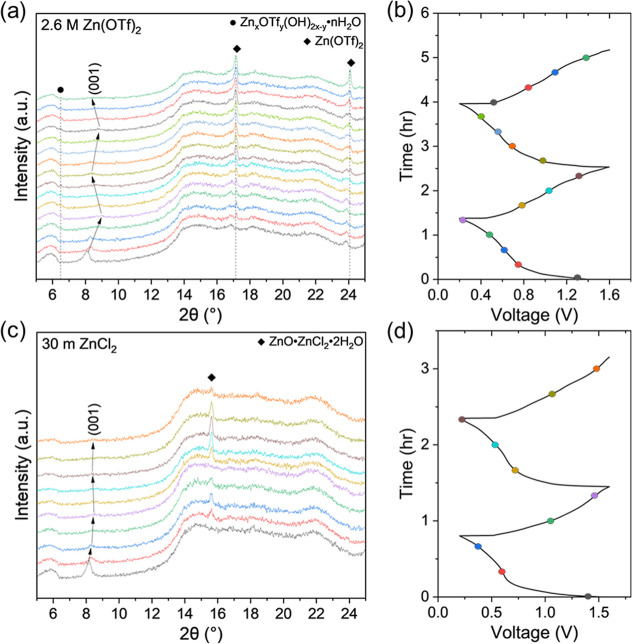
*In situ* XRD patterns and corresponding galvanostatic discharge/charge cycling profiles of aqueous Zn-ion cells containing MD-ZVO electrode and (a,b) 2.6 M Zn­(OTf)_2_ and (c,d) 30 m ZnCl_2_ electrolytes. These cells were cycled at a current density of 0.2 A g^–1^.

The *in situ* XRD patterns of the cell containing 30 m ZnCl_2_ electrolyte (Figure 5c,d) demonstrated fewer changes over two electrochemical discharge/charge cycles compared to the patterns observed for the cell containing 2.6 M Zn­(OTf)_2_. On first discharge, the MD-ZVO (001) peak (highlighted in Figure S7c in Supporting Information) shifted to lower *d*-spacing values from 10.769 Å to 10.591 Å (Δ*d* = 0.178 Å), representing a much smaller contraction of the interlayer region. Interestingly, on first charge, this was followed by a further shift to lower *d*-spacing to 10.443 Å (Δ*d* = 0.148 Å). In the second cycle, the trend in the evolution of the (001) peak position changed: the peak shifted to higher *d*-spacing on discharge and lower *d*-spacing on subsequent charge. Smaller changes in the (001) peak position of MD-ZVO are observed, which is in agreement with the scan rate dependent CV profile behavior. Zn^2+^ ions in the 30 m ZnCl_2_ electrolyte primarily exist as [ZnCl_4_]^2–^ anions, which are unlikely to intercalate into the interlayer region of the negatively charge MD-ZVO layers.^46,61,66^ Additionally, similar to the behavior observed with the cell containing 2.6 M Zn­(OTf)_2_, the MD-ZVO (001) peak in the *in situ* XRD patterns of the cell containing 30 m ZnCl_2_ also decreased in intensity and broadened immediately upon the start of cell operation and remained stable thereafter. This suggests that the same factors of gradual wetting, byproduct precipitation, and MD-ZVO structural disorder evolution also may play a role in the X-ray diffraction signal dampening during *in situ* XRD operation. Additionally, a new peak emerged at 15.62° 2θ, which is attributed to the formation of either a ZnO·ZnCl_2_·2H_2_O phase^67^ or β-Zn­(OH)Cl zincate phase.^68^ Interestingly, these phases are distinct from the commonly observed LDH byproduct in AZIBs containing chloride-based electrolytes, Zn_5_(OH)_8_Cl_2_·H_2_O (ZCH), which is known to decompose into the ZnO·ZnCl_2_·2H_2_O and β-Zn­(OH)Cl phase through thermal decomposition.^67,68^ The applied current during cycling may prevent the ZCH from crystallizing *in situ*, favoring the formation of ZnO·ZnCl_2_·2H_2_O and β-Zn­(OH)Cl phases. Additionally, despite the high 30 m concentration of the ZnCl_2_ electrolyte, no peaks corresponding to pure ZnCl_2_ were observed, suggesting improved concentrated electrolyte stability compared to the 2.6 M Zn­(OTf)_2_ electrolyte.

In conjunction with the *in situ* XRD measurements, *ex situ* XRD analysis was performed on the MD-ZVO electrodes after cycling in cells containing the two electrolytes. XRD patterns of the pristine electrode, electrodes retrieved after the first cycle on discharge down to 0.2 V (0% state of charge or SOC) and on charge up to 1.6 V (100% SOC), and electrodes retrieved on the 100th cycle at 0% and 100% SOC are shown in Figure 6. The *ex situ* XRD patterns reveal the more typical postcycling behavior attributed to vanadium oxides in AZIBs. In the XRD patterns of the electrodes retrieved from the cells containing 2.6 M Zn­(OTf)_2_, two trends observed in the *in situ* XRD measurements were replicated. First, the MD-ZVO (001) peak exhibited a similar change in *d*-spacing and intensity. Some variability in *d*-spacing can arise due to the drying of the ex situ electrodes vs the wetted in situ electrodes. Additionally, the slight intensity reduction of the MD-ZVO (001) peak may similarly arise from structural disorder that is introduced during cycling. The exacerbated intensity reduction can be observed in the XRD pattern of the MD-ZVO electrode obtained at 100% SOC on the 100th cycle, showing the continued evolution of structural disorder over extended cycling. Second, peaks corresponding to the ZTH start to emerge at 0% SOC and completely disappear at 100% SOC, indicating the high reversibility of the LDH formation reaction. The ZTH peaks are additionally more intense in the XRD pattern at the 100th cycle than at the first cycle, showing the growth of larger or more densely packed ZTH crystals with extended cycling. The fact that the ZTH peaks are not at all observed in the 100% SOC peaks, even after 100 cycles, implies that this growth may be electrochemical driven, where the ZTH crystal growth has a lower energetic barrier after many cycles. This suggests that progressive cycling of MD-ZVO electrodes in AZIBs drives the growth of the ZTH byproduct.

**Figure 6:**
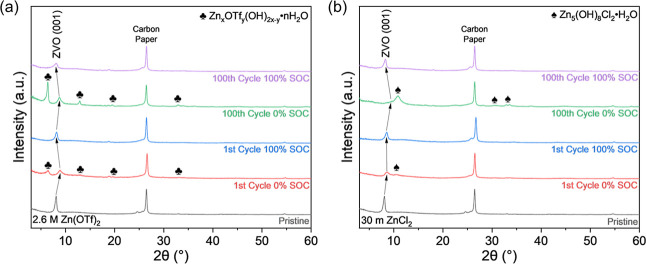
*Ex situ* XRD patterns of MD-ZVO electrodes extracted from aqueous Zn-ion cells containing (a) 2.6 M Zn­(OTf)_2_ and (b) 30 m ZnCl_2_ electrolytes on 1st and 100th galvanostatic discharge/charge cycles after cycling at 1.0 A g^–1^. XRD patterns of the pristine MD-ZVO electrode are shown as a reference in both panels.

Similar details can be observed in the XRD patterns of MD-ZVO electrodes retrieved from cells containing 30 m ZnCl_2_ electrolyte. The same small shift in the MD-ZVO (001) *d*-spacing is exhibited in the XRD patterns during the first cycle; however, the peak shifts become more intense at 100 cycles. While the (001) peak shifted from 10.940 Å to 10.322 Å (Δ*d* = 0.618 Å) on discharge and back to 10.273 Å, Δ*d* = 0.049 Å on the first cycle, the peak positions changed from 9.542 Å at 0% SOC to 10.644 Å at 100% SOC (Δ*d* = 1.102 Å) on the 100th cycle. This drastic change in the (001) *d*-spacing may represent the expansion of the MD-ZVO layers when the cells are left to cycle for extended periods, possibly enabled by more intercalation of Zn^2+^ ion species like Zn^2+^ and [ZnCl]^+^ in conjunction with water infiltration from the solvated Zn^2+^ species.^61^ Another difference between the *in situ* and *ex situ* XRD patterns is the formation of peaks attributed to ZCH.^47^ Over extended cycling, the ZCH peaks become more intense at 0% SOC from the first cycle to the 100th cycle, while disappearing completely on subsequent charge steps at 100% SOC, showing similar high reversibility and growth of ZCH crystals that was observed on the electrodes cycled with 2.6 M Zn­(OTf)_2_. The presence of ZCH shows the mixed Zn^2+^/H^+^ charge storage mechanism for the 30 m ZnCl_2_ electrolyte. The evolution of the ZTH in cells containing 2.6 M Zn­(OTf)_2_ and ZCH in cells containing 30 m ZnCl_2_ electrolytes indicate the reversible growth of the byproducts over extended cycling.

The evolution of the byproduct formation was further examined using *ex situ* SEM imaging (Figure 7) and EDS mapping (Figures S9–S11 in Supporting Information). The surface of the pristine MD-ZVO electrode contains primarily MD-ZVO and carbon black particles (Figure 7a), and these two phases are easily distinguished by the EDS elemental mapping (Figure S9 in Supporting Information) as the V, O, and Zn maps are distinct from the C map. In cells containing the 2.6 M Zn­(OTf)_2_ electrolyte, after the initial discharge to 0% SOC (Figure 7b), the electrode surface shows noticeable morphology changes, with small flakes, corresponding to the ZTH phase according to the XRD analysis, appearing scattered across the surface. Upon subsequent charging to 100% SOC (Figure 7c), the surface morphology appears to return to its pristine state, with no significant presence of ZTH detected. This behavior is consistent with the changes observed in both *in situ* and *ex situ* XRD patterns, indicating the reversible formation and dissolution of the ZTH byproduct layer. After 100 electrochemical cycles, the electrode retrieved from the cell stopped at 0% SOC (Figure 7d) shows surface flakes that are noticeably larger than those observed after the first discharge. Upon subsequent charging to 100% SOC (Figure 7e), the electrode surface again appears pristine, replicating the trend observed with the electrodes extracted during the first cycle. The growth of the ZTH crystals on the surface of the MD-ZVO electrodes is highly reversible, yet the crystal size progressively increases with extended cycling. From ex situ EDS mapping of the electrode surface (Figure S10 in Supporting Information), the S signal indicates the presence of the ZTH crystals. Not only can the S signal be observed at 100% SOC after 100 cycles, but some S signal is also seen at 100% SOC during the first cycles, suggesting that miniscule amounts of ZTH are present at all times during initial cycling that may serve as the seed crystals for ZTH growth in later cycles leading to formation of larger crystals observed through SEM imaging (Figure 7d). The electrodes retrieved from the cells containing the 30 m ZnCl_2_ electrolyte exhibit a similar reversible byproduct formation pattern. Upon initial discharge to 0% SOC (Figure 7f), vertically oriented stacked flakes of ZCH form nodes on the surface that disappear on subsequent charge to 100% SOC (Figure 7g). These flakes appear to grow much larger after 100 cycles at 0% SOC (Figure 7h) before again disappearing on subsequent charge (Figure 7i). However, *ex situ* EDS mapping of electrodes retrieved from the cells containing 30 m ZnCl_2_ reveals the presence of Cl at 100% SOC not only after the first cycle, but also after 100 discharge/charge cycles, suggesting that nucleation sites for ZCH growth persist upon charging and continue to accumulate over extended cycling. Additionally, at 100% SOC, the Cl signals for the electrodes obtained from ZnCl_2_-containing cells are stronger than the S signals of the electrodes from Zn­(OTf)_2_-containing cells, indicating the poorer reversibility of ZCH compared to ZTH. Therefore, our *ex situ* imaging analysis indicates that after forming on discharge, the byproduct phases largely decompose upon charging, while retaining small ZTH/ZCH seeds that act as nucleation sites for growth of larger byproduct crystals on discharge in subsequent discharge/charge cycles.

**Figure 7:**
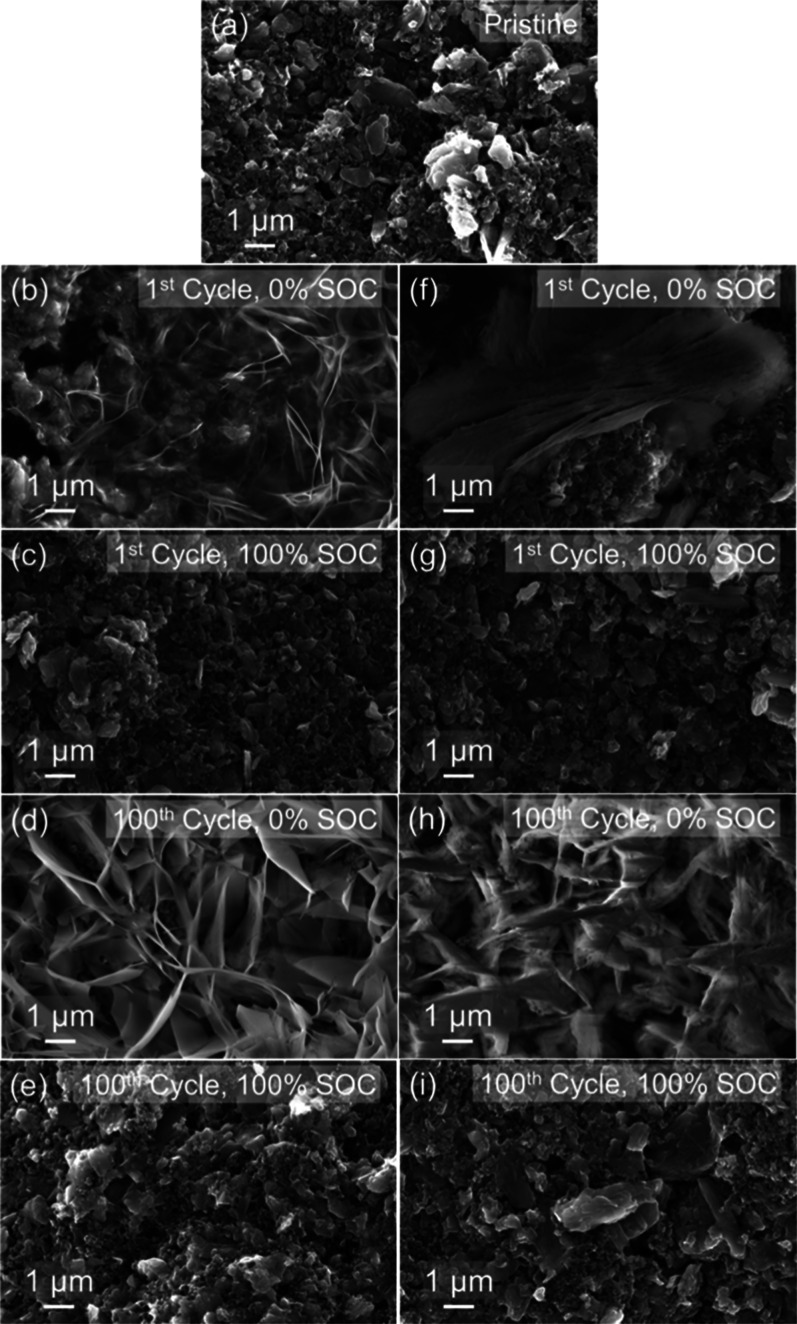
SEM images of the (a) pristine MD-ZVO electrode and (b–i) MD-ZVO electrodes extracted from aqueous Zn-ion cells containing (b–e) 2.6 M Zn­(OTf)_2_ and (f–i) 30 m ZnCl_2_ electrolytes. SEM images were taken after the cells were stopped at (b,f) 1st cycle, 0% SOC, (c,g) 1st cycle, 100% SOC, (d,h) 100th cycle, 0% SOC, and (e,i) 100th cycle, 100% SOC.

To provide a more detailed assessment of the surface composition, *ex situ* XPS measurements were carried out on electrodes extracted from the cells containing 2.6 M Zn­(OTf)_2_ and 30 m ZnCl_2_ (Figure 8). Analysis of the V 2p region in the XPS spectrum of the MD-ZVO powder (Figure S12 in Supporting Information) indicated a mixed oxidation state of vanadium, with V^5+^ (V 2p_3/2_: 5172 eV, V 2p_1/2_: 524.5 eV) and V^4+^ (V 2p_3/2_: 515.8 eV, V 2p_1/2_: 523.2 eV). This mixed V^4+/5+^ state likely results from the preintercalated Zn^2+^ ions and is present in a ratio of 79.3% V^5+^ to 20.7% V^4+^. Analysis of the O 1s region revealed two peaks corresponding to the O–V bonds (O 1s: 529.0–530.0 eV) from the MD-ZVO lattice and O–H bonds (O 1s: ∼531 eV) from the interlayer water molecules. The shape of the V 2p and O 1s regions shows minimal changes when the MD-ZVO powder was mixed with carbon black and PTFE and processed into an electrode (Figure 8a), with an observed change in the V^5+^/V^4+^ ratio to 82.1%/17.9%. These similarities agree with the observations made during *in situ* XRD cell fabrication (Figure S7a in Supporting Information). For the cells containing 2.6 M Zn­(OTf)_2_, after initial discharge to 0% SOC (Figure 8b), the V 2p signal in the range of 513–527 eV appears to almost disappear while the O–H peak becomes the dominant feature in this region of the XPS spectrum. The intensification of the O–H peak, coupled with the emergence of a peak at ∼532 eV that was attributed to O–S bonds, indicates the formation of the ZTH byproduct on the surface of the electrode that suppresses the signal detected from the active MD-ZVO phase in the electrode. The V^5+^/V^4+^ ratio also decreases to 66.8/33.2%, in agreement with the intercalation of Zn^2+^ and protons, which reduce vanadium during electrochemical cycling. On subsequent charge to 100% SOC (Figure 8c), the XPS spectra returns to a similar state as the pristine electrode, however marked by a change in the presence of the O–H peak and relatively more intense O–S peak that is indicative of residual ZTH. The persistence of the O–S peak after charging to 100% SOC follows the trend established with *ex situ* EDS mapping, where S was observed on the surface of the MD-ZVO electrode in small quantities, which would suggest some irreversibility in the ZTH formation/dissolution reaction. Additionally, the V^5+^/V^4+^ ratio at 100% SOC is 80.5%/19.5%, similar to that of the pristine electrode, indicating efficient extraction of the cations intercalated during the discharge step. The electrodes extracted from the cells after 100 electrochemical cycles demonstrate a similar trend of XPS spectra evolution, with the emergence of the O–H peak and the suppression of the V 2p region at 0% SOC (Figure 8d) and the recovery of the original spectra shape at 100% SOC (Figure 8e). The O–S peak appears to increase in relative intensity in both states after 100 cycles, which can be attributed to the larger ZTH growth observed in *ex situ* SEM imaging of the electrode after 100 cycles as compared to the electrode initially discharged to 0% SOC (Figure 7d), as well as the presence of residual ZTH crystals on the MD-ZVO electrode surface after 100 cycles (Figure 7e). The decreased V^5+^/V^4+^ ratio at 0% SOC (69.4%/30.6%) and its subsequent increase at 100% SOC (86.8%/13.2%) on the 100th cycle confirms efficient reversibility of electrochemically cycled ions (Zn^2+^ ions and protons) over extended cycling.

**Figure 8:**
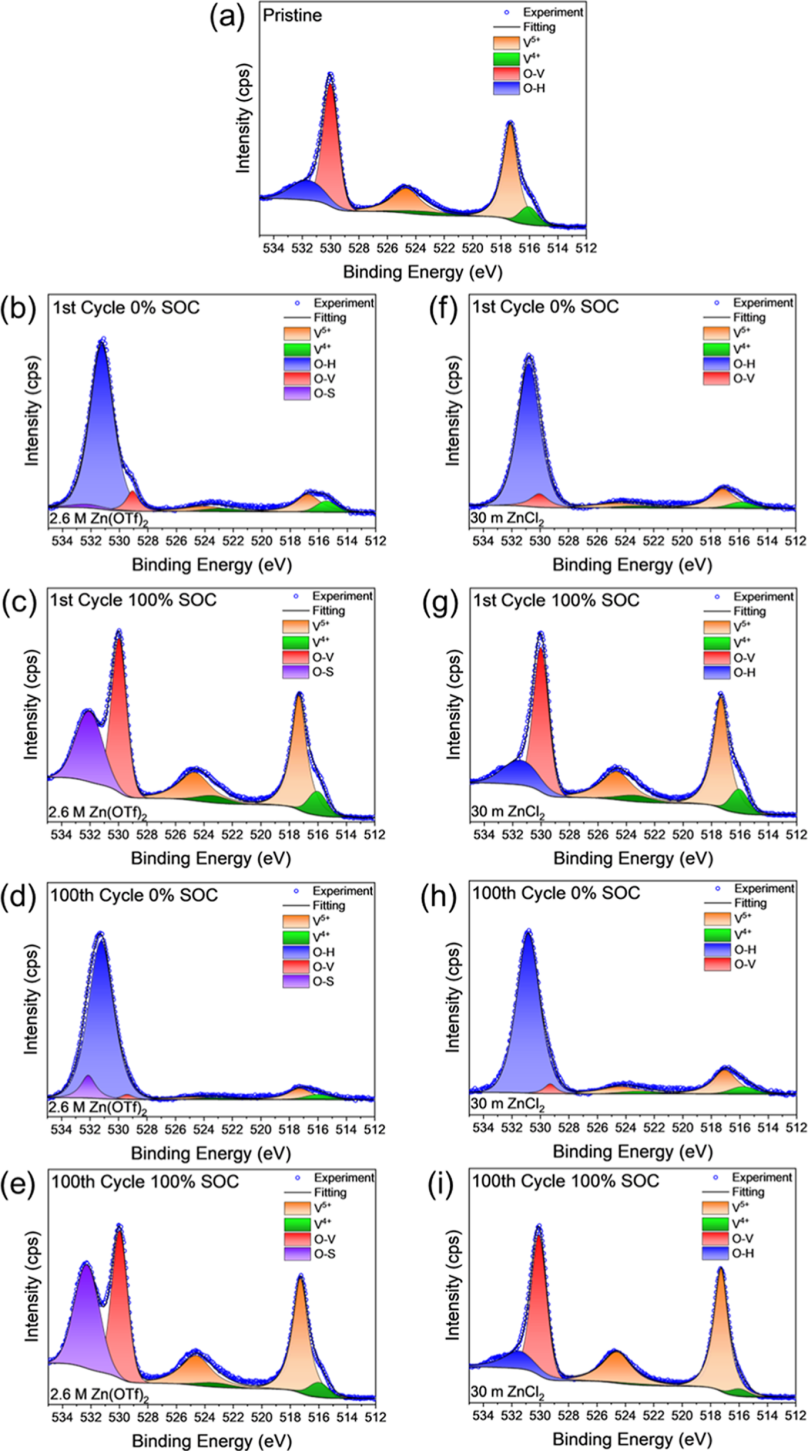
XPS analysis of MD-ZVO electrodes: (a) pristine electrode and (b–i) electrodes obtained from cells containing (b-e) 2.6 M Zn­(OTf)_2_ and (f–i) 30 m ZnCl_2_ electrolytes. The electrodes were retrieved from cells stopped at (b,f) 1st cycle, 0% SOC, (c,g) 1st cycle, 100% SOC, (d,h) 100th cycle, 0% SOC, and (e,i) 100th cycle, 100% SOC.

Many of the same trends in the XPS spectra evolution can be observed for the MD-ZVO electrodes cycled in cells containing 30 m ZnCl_2_. The first cycle discharge to 0% SOC (Figure 8f) features a similar intensification of the O–H peak while the V 2p region is suppressed, followed by the recovery of the spectral shape upon charging to 100% SOC (Figure 8g), indicative of the growth and dissolution of the ZCH layer on the MD-ZVO electrode surface. While no equivalent O–Cl peak was observed in the O 1s region, the small change in the V^5+^/V^4+^ ratio from 76.5%/23.5% at 0% SOC to 81.1%/18.9% at 100% SOC is consistent with the more proton-dominant charge storage properties of the 30 m ZnCl_2_ electrolyte, which was observed in the scan rate dependent CV profile evolution. After 100 cycles, the MD-ZVO electrodes cycled using 30 m ZnCl_2_ share a similar trend to the electrodes cycled using 2.6 M Zn­(OTf)_2_, where the V^5+^/V^4+^ ratios change from 76.8%/23.2% at 0% SOC (Figure 8h) to 93.6%/6.4% at 100% SOC (Figure 8i). The similar oxidation states present after initial discharge and discharge after 100 cycles suggest a similar depth of discharge, but the higher oxidation state on subsequent charge may indicate that the role of Zn^2+^ ion intercalation becomes more prominent in cells containing 30 m ZnCl_2_ with extended cycling. Additionally, the larger change in the V^5+^/V^4+^ ratio observed from the electrodes extracted from cells containing 2.6 M Zn­(OTf)_2_ compared to electrodes extracted from cells containing 30 m ZnCl_2_ may also indicate the larger contribution of Zn^2+^ charge storage from use of Zn­(OTf)_2_ electrolytes, in agreement with the *in situ* and *ex situ* XRD analysis. This agrees with the extended cycle life analysis that shows lower capacities for cells containing 30 m ZnCl_2_ compared to cells with 2.6 M Zn­(OTf)_2_.

Electrochemical impedance spectroscopy (EIS) spectra of cells containing 2.6 M Zn­(OTf)_2_ and 30 m ZnCl_2_ electrolytes that were cycling over 100 cycles at 1.0 A g^–1^ (Figure S13 in Supporting Information) further detailed the differences in electrochemical charge storage behavior between the two electrolytes. Given uncertainties in the nature of the charge-carrying species (Zn^2+^ ions and protons), we focused our analysis of EIS spectra on evaluating the charge transfer resistance from the high-frequency region. The progression of EIS plots from the first cycle to the 100th cycle shows two distinct sets of changes related to the nature of the electrolyte. In the cell containing 2.6 M Zn­(OTf)_2_, the EIS plot at OCV (Figure S13a in Supporting Information) shows a relatively large semicircle with a short tail indicative of a significant charge-transfer resistance (*R*_ct_) and low diffusion resistance, typical of a Randles circuit model (Figure S14a in Supporting Information). Fitting the EIS spectrum obtained at the open circuit voltage (OCV) to this model (Table S2, Figures S15 and S16 in Supporting Information) confirms a value for *R*_ct_ of 2025 Ω. After the first discharge step to 0% SOC, the semicircle shrank resulting in a fitted *R*_ct_ value of 217.9 Ω, which agrees with the intercalation of Zn^2+^ ions and protons that increase the conductivity of the MD-ZVO. Additionally, the high-frequency region at 0% SOC showed a good fit to a combination of two semicircles, where the second semicircle could be attributed to the formation of a byproduct layer on the surface of the electrode represented by a resistor and capacitor in parallel in the equivalent circuit model (Figure S14b in Supporting Information). This observation agrees with the formation of the ZTH layer on the surface of the MD-ZVO electrode. Upon subsequent charging to 100% SOC, the semicircle contraction indicates a decrease of the charge transfer resistances, which can be attributed to the decomposition of the ZTH byproduct layer during charging. Additionally, the further reduction in *R*_ct_ to 26.4 Ω after ion extraction indicates a difference in the electrolyte–electrode surface characteristics as compared to the state of the cell at OCV before cycling. This behavior has been previously reported for vanadium oxide electrodes and has been attributed to residual Zn^2+^ ions that remain in the vanadium oxide structure after cycling.^69^ The trend of the semicircle evolution in EIS spectra with the emergence of an additional semicircle at 0% SOC and subsequent disappearance at 100% SOC was also observed in the Nyquist plots for the 10th (Figure S13b in Supporting Information) and 100th cycles (Figure S13c in Supporting Information). Interestingly, while the curves at 100% SOC appear to stay consistent across the three chosen cycles, the 0% SOC curves evolve through the 100-cycle duration. The shape of the EIS spectra in the high frequency region at 0% SOC initially appears as two visually distinct semicircles which appear to overlap in later cycles, indicating the larger contribution of the ZTH layer to interfacial resistance over extended cycling.

For the cell containing the 30 m ZnCl_2_ electrolyte, there is significantly less noticeable evolution of the EIS spectra over 100 discharge/charge cycles compared to the cell containing the 2.6 M Zn­(OTf)_2_ electrolyte. The EIS spectrum obtained at OCV (Figure S13d in Supporting Information) appears at relatively low impedances compared to the spectrum exhibited by the 2.6 M Zn­(OTf)_2_-containing cell. The lower *R*_ct_ value of 4.9 Ω at OCV indicates an improved electrolyte–electrode interface, likely enabled by improved wetting and smaller size of protons as the dominant charge carriers in the 30 m ZnCl_2_ electrolyte.^61,70^ After the first discharge to 0% SOC, the semicircle area shifted to lower impedance values and expanded horizontally. This resulted in a good fit to two semicircles, reflecting a behavior similar to that observed in the 2.6 M Zn­(OTf)_2_ electrolyte, in agreement with the intercalation of protons and Zn^2+^ ions (one semicircle) as well as the formation of the ZCH byproduct layer (second semicircle). The larger capacitance and resistance values of the second semicircle, determined through the fitting to the equivalent circuit, in the spectra obtained from the 30 m ZnCl_2_-containing cell compared to the 2.6 M Zn­(OTf)_2_-containing cell (Table S2 in Supporting Information) suggests the ZCH byproduct layer is more insulating than the ZTH layer which may contribute to the more limited charge storage of MD-ZVO in 30 m ZnCl_2_ over extended cycling compared to the performance of MD-ZVO in 2.6 M Zn­(OTf)_2_. Upon subsequent charge to 100% SOC, the EIS spectrum closely resembles the one recorded at OCV, supporting the high reversibility of the discharge and charge cycles. The formation of the second semicircle, followed by the disappearance of this feature and the recovery of the EIS spectrum shape is replicated in the spectra obtained after 10 (Figure S13e in Supporting Information) and 100 cycles (Figure S13f in Supporting Information), further supporting high reversibility with extended cycling.

The agreement between the *in situ* and *ex situ* analyses with the rate capability and cycle life testing indicates that the electrochemical formation of byproducts during MD-ZVO electrodes cycling with aqueous Zn-ion electrolytes is reflected in both performance metrics and the underlying reaction mechanism. The dynamics of charge storage in aqueous zinc-ion batteries rely on the understanding of charge carriers present and the properties and reactivity of the selected electrolytes. From the initial screening of electrolytes, compositions containing bulkier anions such as 2.6 M Zn­(OTf)_2_ or those that were highly concentrated like 30 m ZnCl_2_ demonstrated higher stability during extended cycling of MD-ZVO electrodes. The good performance of these electrolytes is attributed to the suppression of side reactions involving water, which, although beneficial for ion transport in some cases, is the primary cause of vanadium oxide dissolution. *In situ* and *ex situ* characterization of MD-ZVO electrodes revealed the formation of layered double hydroxides and other byproducts on the electrode surface, which contribute to the electrochemical response and may influence the long-term stability of the MD-ZVO electrode in AZIBs. While the byproduct layer initially appeared to be highly reversible, analysis after 100 cycles indicated that a small amount of byproduct remained on the electrode surface and may have served as nucleation sites for the growth of larger byproduct particles in later cycles. This was particularly evident in cells containing 30 m ZnCl_2_, where the lower reversibility of ZCH could explain the faster capacity decay over the first 100 cycles as compared to cells with 2.6 M Zn­(OTf)_2_. However, despite the more efficient byproduct decomposition observed over 100 cycles with 2.6 M Zn­(OTf)_2_, long-term cycling over 1000 cycles suggested that the stability characteristics reversed, with the cells containing the 30 m ZnCl_2_ electrolyte showing excellent stability after the 500th cycle. These findings demonstrated the potential of 30 m ZnCl_2_ to serve as a highly stable electrolyte, making it especially attractive for applications that require extreme cycle life. In addition to the choice of highly concentrated electrolytes, the high performance of MD-ZVO was attributed to its distinctive nanoflower morphology. On one hand, with the 2.6 M Zn­(OTf)_2_ electrolyte, the MD-ZVO electrodes exhibited some of the highest capacities compared to other Zn-preintercalated vanadium oxides (Table S3 in Supporting Information) and other MXene-derived vanadium oxides (Table S4 in Supporting Information). On the other hand, the lower capacities demonstrated by the cells with the 30 m ZnCl_2_ electrolyte can be compensated by the lower cost of ZnCl_2_, making it a promising alternative to typical electrolytes for large-scale applications. Furthermore, highly concentrated electrolytes appear to be highly promising for extending the voltage window of AZIBs and providing unprecedented stability despite the inherent instability of water-based systems.^71,72^ Our findings emphasize that the development of next-generation aqueous Zn-ion batteries depends primarily on designing highly performing cathode materials, followed by optimizing electrolyte compositions that synergize effectively with these cathodes.

## 4 Conclusions

This study demonstrates the excellent performance of V_2_CT_*x*_ MXene-derived Zn-preintercalated bilayered vanadium oxide (MD-ZVO) in aqueous Zn-ion batteries using highly concentrated 2.6 M Zn­(OTf)_2_ and 30 m ZnCl_2_. MD-ZVO with a nanoflower morphology was synthesized for the first time using V_2_CT_*x*_ MXene, yielding 2D nanosheets with a composition of Zn_0.19_V_2_O_5_·0.57H_2_O and a (001) *d*-spacing of 10.753 Å. Cycling of MD-ZVO electrodes revealed the high initial capacities and improved stability over 350 cycles of saturated 2.6 M Zn­(OTf)_2_ (450 mAh g^–1^, 58% capacity retention) and highly concentrated 30 m ZnCl_2_ (315 mAh g^–1^, 59% capacity retention) over dilute 2 M ZnSO_4_ (337 mAh g^–1^, 9% capacity retention) and 2 M ZnCl_2_ (397 mAh g^–1^, 13% capacity retention) when cycled at a current rate of 0.1 A g^–1^. The improved stability was attributed to the solvation structure and the lower water activity of the highly concentrated electrolytes, suppressing the dissolution of the vanadium oxide cathode. Scan rate dependent CV cycling, rate capability, and extended cycling (1000 cycles at 1.0 A g^–1^) revealed the notable contribution of Zn^2+^ species in 2.6 M Zn­(OTf)_2_ and the proton dominant behavior of 30 m ZnCl_2_. *In situ* and *ex situ* XRD confirmed the reversible intercalation and byproduct formation processes, where the nature of reversibly intercalating cations was determined by the shifting of the MD-ZVO (001) peak and the reversible formation of Zn_*x*_OTf_*y*_(OH)_2*x*–*y*_·*n*H_2_O (ZTH) in cells containing 2.6 M Zn­(OTf)_2_ and Zn_5_(OH)_8_Cl_2_·H_2_O (ZCH) in cells containing 30 m ZnCl_2_ was revealed by the emergence of new XRD peaks during discharge. *Ex situ* SEM, EDS, and XPS revealed the highly reversible surface ZTH and ZCH crystallization and seed formation, with larger byproduct crystals appearing after extended cycling. EIS fitting further confirmed the growth of these byproduct layers, as evidenced by a second high-frequency semicircle. These results demonstrate the excellent performance of MD-ZVO electrodes in aqueous Zn-ion batteries, as well as the vital role of electrolyte selection for high performance of MXene-derived oxides for AZIBs. Choosing cathode materials that display superior energy storage properties must be paired with compatible electrolyte compositions to fully realize next-generation aqueous Zn-ion batteries.

## Supplementary Material



## Data Availability

The data underlying this study are openly available in Materials Commons at 10.13011/m3-ssfk-ts03.

## Notes

Images of *in situ* XRD cell; XRD patterns and SEM images of V_2_AlC MAX phase and V_2_CT_*x*_ MXene precursors; additional SEM images of MD-ZVO; EDS spectrum and compositional analysis of MD-ZVO; AAS absorbance plots for MD-ZVO; full Raman spectrum of MD-ZVO; table of peak positions from CV profiles; additional *in situ* XRD cycling data; XRD pattern of Zn­(OTf)_2_ powder; SEM image and EDS mappings of pristine MD-ZVO electrode; SEM images and EDS mappings of MD-ZVO electrodes cycled in 2.6 M Zn­(OTf)_2_ electrolyte; SEM images and EDS mappings and MD-ZVO electrodes cycling in 30 m ZnCl_2_ electrolyte; XPS spectrum of MD-ZVO; EIS spectra of cells containing MD-ZVO with 2.6 M Zn­(OTf)_2_ and ZnCl_2_; equivalent circuit models used for EIS fitting; table of circuit element values obtained from EIS spectra fitting; resulting EIS spectra from fitting; summary of electrochemical cycling parameters and performance of Zn-preintercalated vanadium oxide cathodes in aqueous Zn-ion cells reported in literature and obtained in this study; summary of electrochemical cycling parameters and performance of MXene-derived vanadium oxide cathodes in aqueous Zn-ion cells reported in literature and obtained in this study (PDF)
