# Supplementary material for: Operation of MXene-Derived Zinc-Preintercalated Bilayered Vanadium Oxide Cathode in Aqueous Zn-Ion Batteries
Source: ACS Appl Energy Mater. 2025 Aug 27;8(17):12695–711. doi: 10.1021/acsaem.5c01721 (PMC12421514; doi:10.1021/acsaem.5c01721)
Supplement: Supplementary file 1 [file ae5c01721_si_001.pdf]

SUPPORTING INFORMATION

Operation of MXene-Derived Zinc-Preintercalated Bilayered Vanadium Oxide Cathode in Aqueous Zn-Ion Batteries

Timofey Averianov[1], Kyle Matthews[2], Xinle Zhang[1], Huyen T.K. Nguyen[1,2], Yuan Zhang[2], Yury Gogotsi[2] , Ekaterina Pomerantseva[1*]

[1] Materials Electrochemistry Group, Department of Materials Science and Engineering, Drexel University, Philadelphia, Pennsylvania 19104, United States

[2] A.J. Drexel Nanomaterials Institute Department of Materials Science and Engineering, Drexel University, Philadelphia, Pennsylvania 19104, United States

* Corresponding Author: ep423@drexel.edu

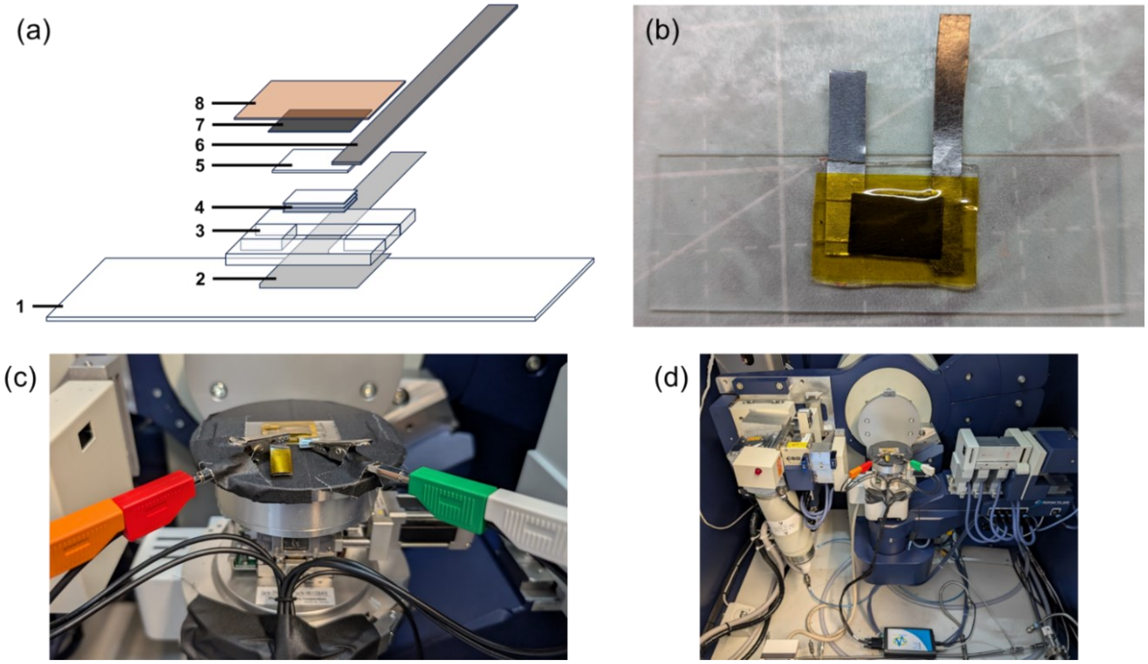

Figure S1. Custom-built *in situ* XRD cell assembly and setup. (a) Schematic illustration of *in situ* XRD cell consisting of (1) glass microscope slide (Fisher Scientific), (2) Zn metal foil anode (MTI Corporation), (3) VHB 4910 tape cell body (3 M™), (4) glass fiber separator (Whatman), (5) polypropylene separator (Celgard), (6) graphite foil (Fisher Scientific), (7) working electrode coated on carbon paper, (8) Kapton film; (b) digital photograph of *in situ* XRD cell; (c) digital photograph of the *in situ* XRD cell and potentiostat leads attached onto XRD sample holder; (d) digital photograph of the full setup for *in situ* XRD testing of MD-ZVO cathodes in aqueous Zn-ion batteries (AZIBs) inside the diffractometer.

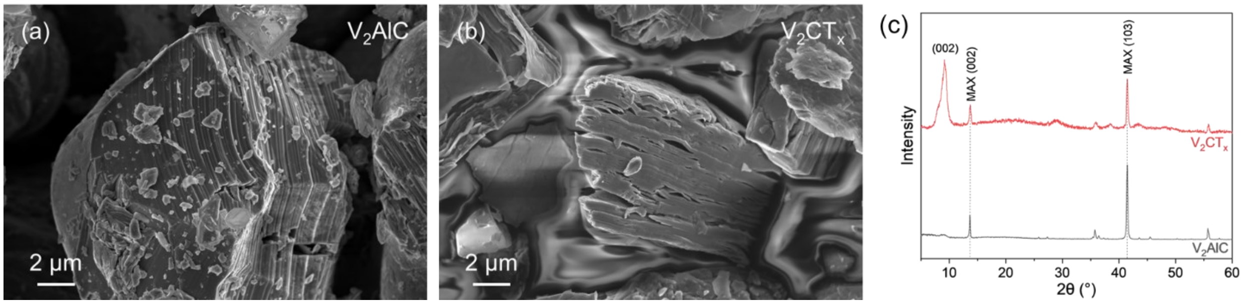

Figure S2. SEM images of (a) V_2AlC MAX phase powder and (b) V_2CT_x MXene multilayer nanoflakes; (c) XRD patterns of the MAX phase and MXene precursors.

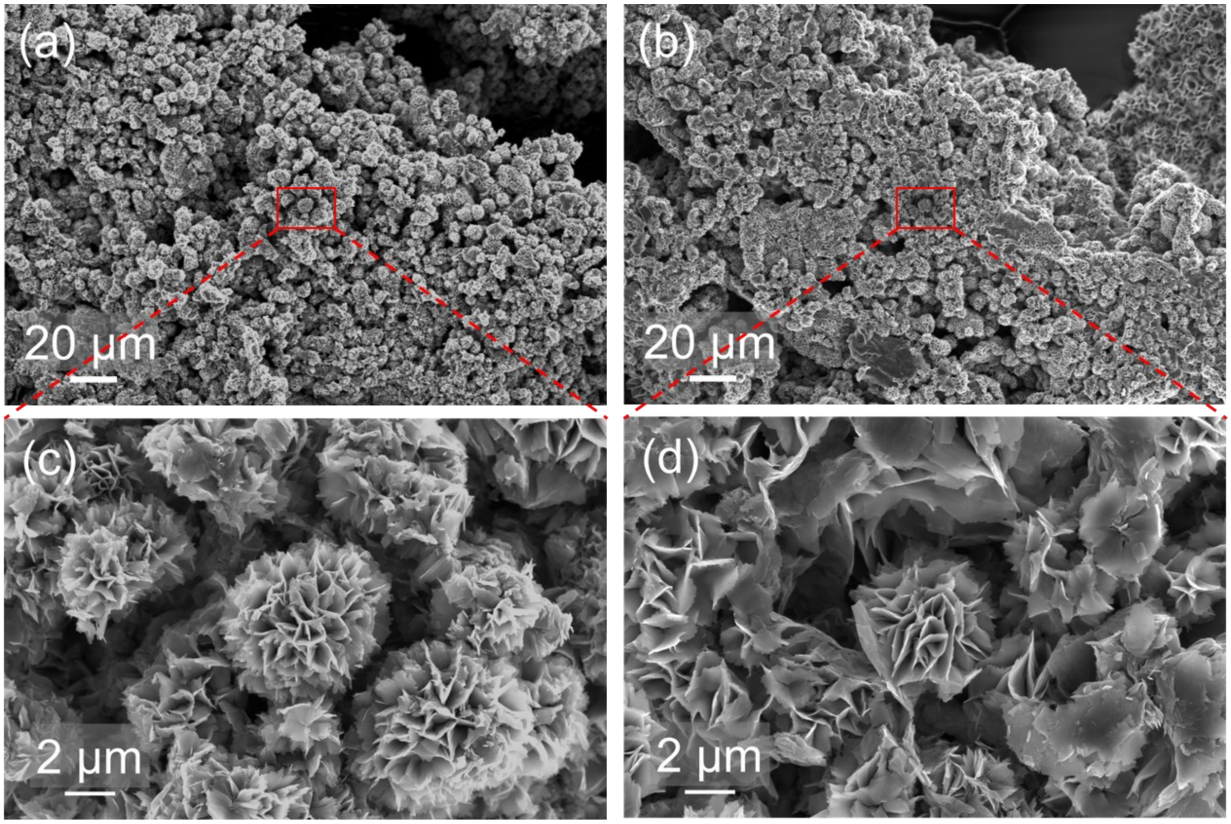

Figure S3. Additional (a,b) low and (c,d) high magnification SEM images of MD-ZVO. (a,c) and (b,d) are taken from two different batches of the synthesized MXene-derived oxide.

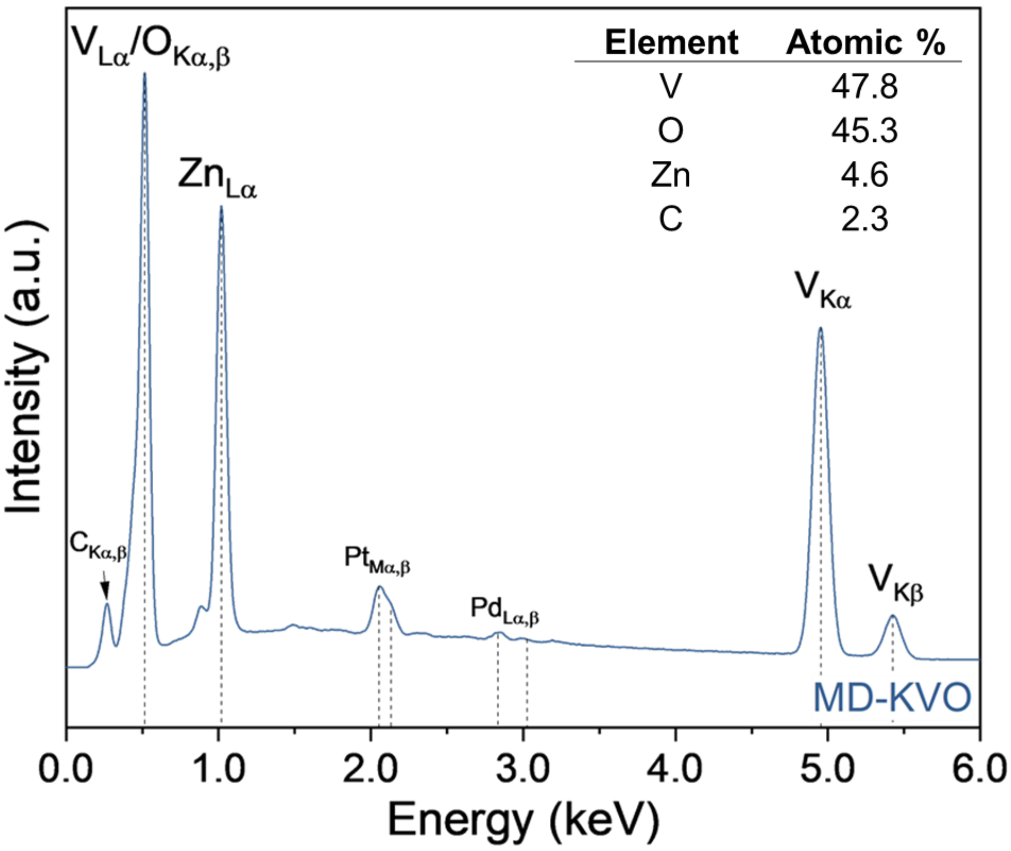

Figure S4. EDS spectra of MD-ZVO with compositional analysis for V, O, Zn, and C. Pt and Pd are present due to sputter coating.

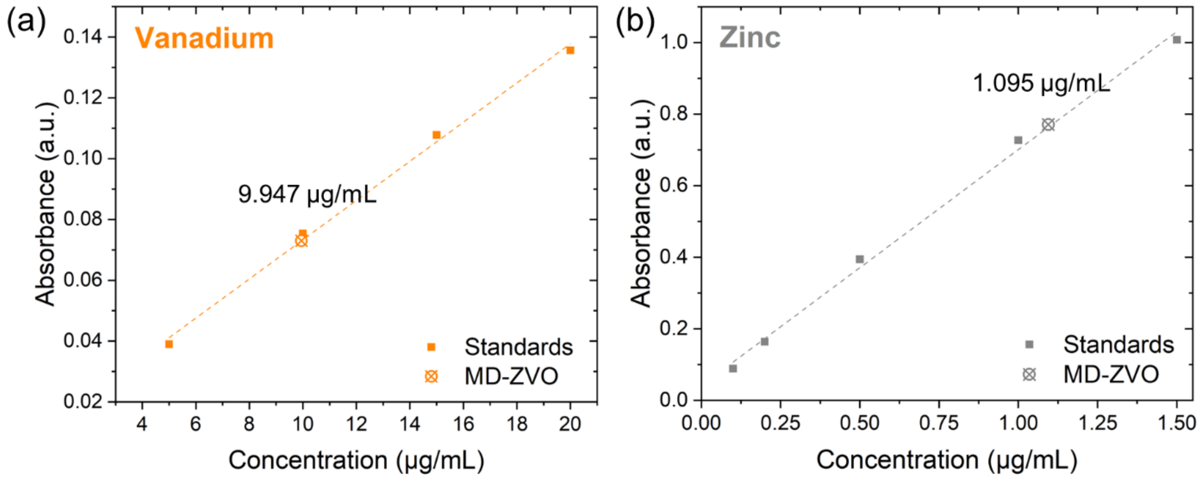

Figure S5. AAS absorbance plots for (a) vanadium and (b) zinc.

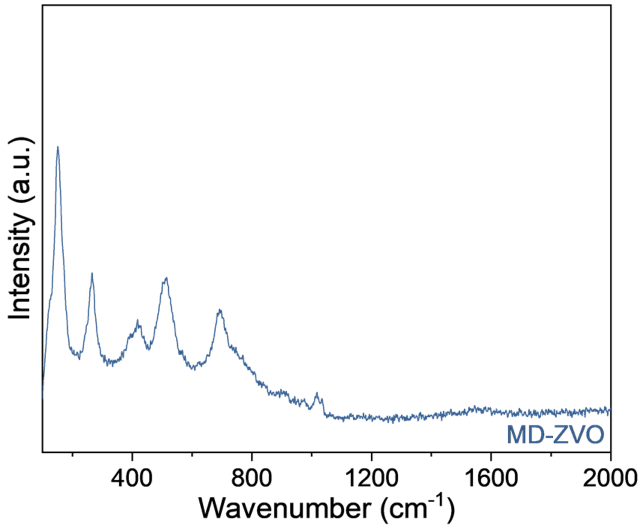

Figure S6. Full Raman spectrum of MD-ZVO.

Table S1. Peak positions for CV profiles from **Figure 3**.

2 M $ZnSO_4$		2.6 M $Zn(OTf)_2$		2 M $ZnCl_2$		30 m $ZnCl_2$	
Discharge	Charge	Discharge	Charge	Discharge	Charge	Discharge	Charge
1.286	1.403	1.358	1.438	1.306	1.460		
		1.338	1.374	1.195	1.239		
1.135	1.267	1.177	1.213				
		1.127	1.144				
0.933	1.206	0.977	1.066	0.972	1.161	1.072	1.172
0.816	1.158	0.792	0.995	0.854	1.082	0.926	1.013
0.568	0.832	0.620	0.738	0.624	0.758	0.652	0.807
0.401	0.647	0.440	0.558	0.491	0.653	0.532	0.656

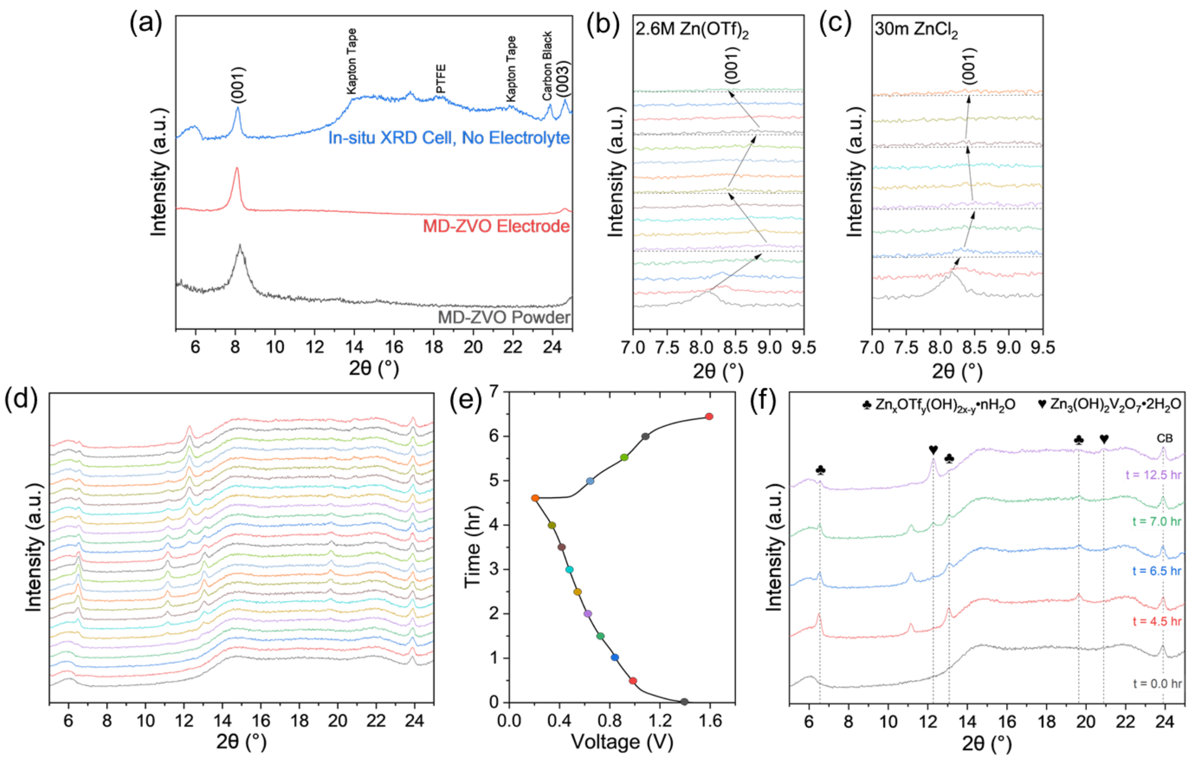

Figure S7. (a) XRD patterns of MD-ZVO as it is processed into an electrode and used in the *in situ* XRD cell. (b,c) Zoomed-in (001) region of **Figure 5a** and **c**. Dotted lines were added below selected patterns to better illustrate location of (001) peak. (d) in-situ XRD patterns and (e) cycling profile for $Zn(OTf)_2$-containing cell cycled at 90 mA g^{-1} which was stopped after 1 cycle; XRD patterns continued to be collected after cell operation was stopped. (f) XRD patterns from cell at selected times.

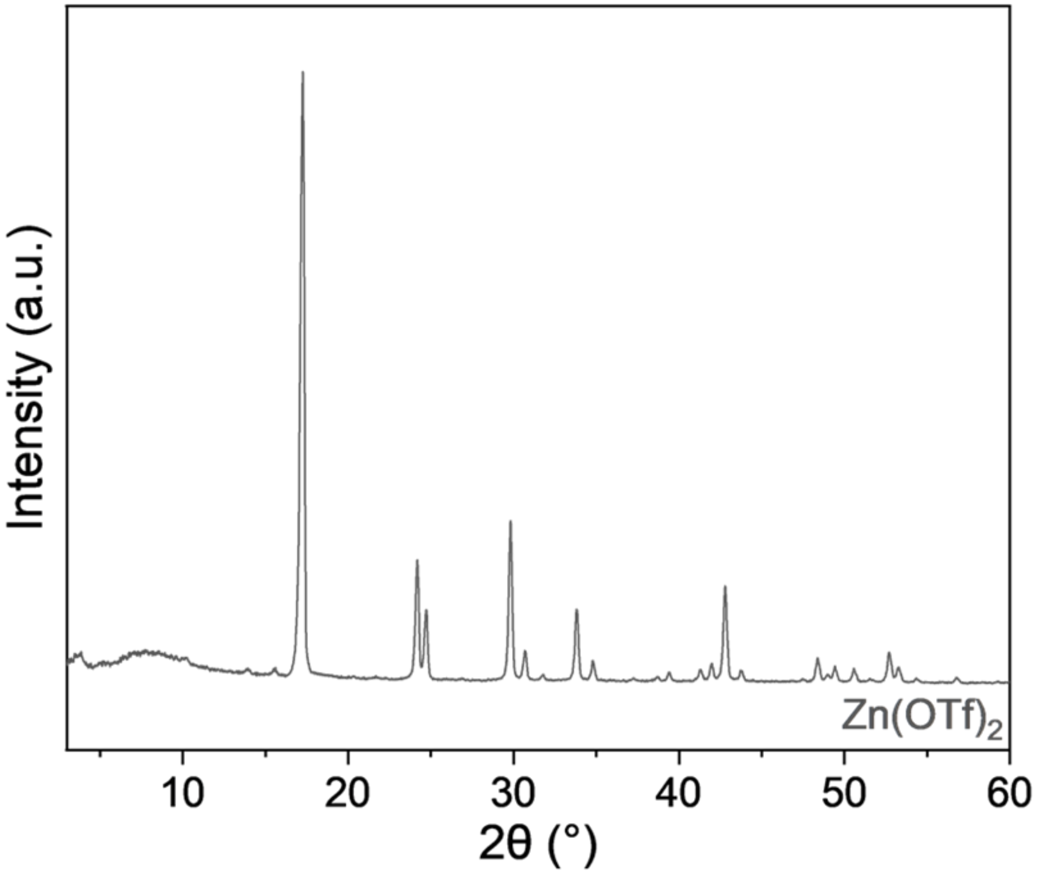

Figure S8. XRD pattern of $Zn(OTf)_2$ powder.

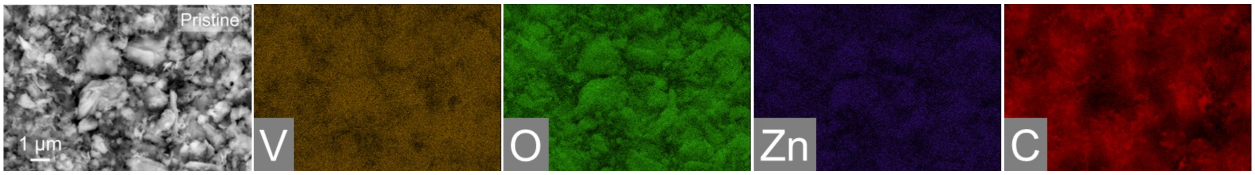

Figure S9. SEM image and EDS maps of V, O, Zn, and C taken for the pristine MD-ZVO electrode. The V, O, and Zn maps correspond to the MD-ZVO active material while the C map corresponds to the carbon black additive. The Zn/V ratio calculated from these EDS mappings is 0.114.

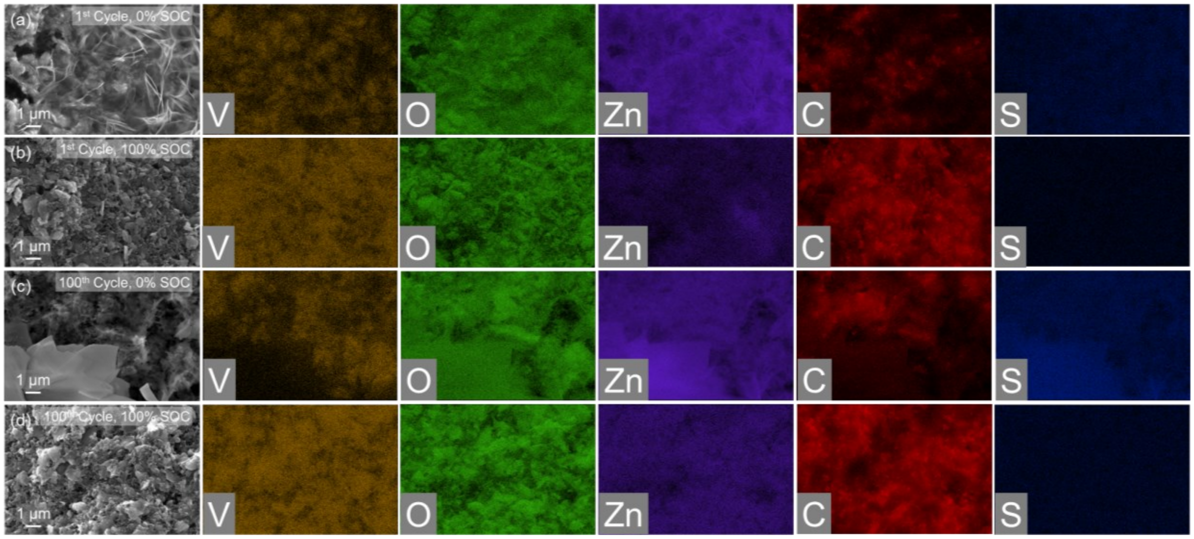

Figure S10. SEM images and EDS maps of V, O, Zn, C, and S taken for the MD-ZVO electrodes retrieved from AZIB cells containing the 2.6 M $Zn(OTf)_2$ electrolyte at (a) 1st cycle 0% SOC (Zn/V ratio = 1.684), (b) 1st cycle 100% SOC (Zn/V ratio = 0.167), (c) 100th cycle 0% SOC (Zn/V ratio = 2.434), and (d) 100th cycle 100% SOC (Zn/V ratio = 0.196). At 0% SOC, the surface becomes coated with ZTH flakes, which can be correlated with more intense O, Zn, and S maps. At 100% SOC, these flakes dissolve, leaving the electrode surface mostly pristine. S mapping of 100% SOC electrodes does show residual S that indicated some undissolved ZTH that may serve as seed crystals for ZTH formation during discharge. The elevated Zn/V ratios for electrodes retrieved from 100th cycle compared to 1st cycle also implies residual ZTH on electrode surface.

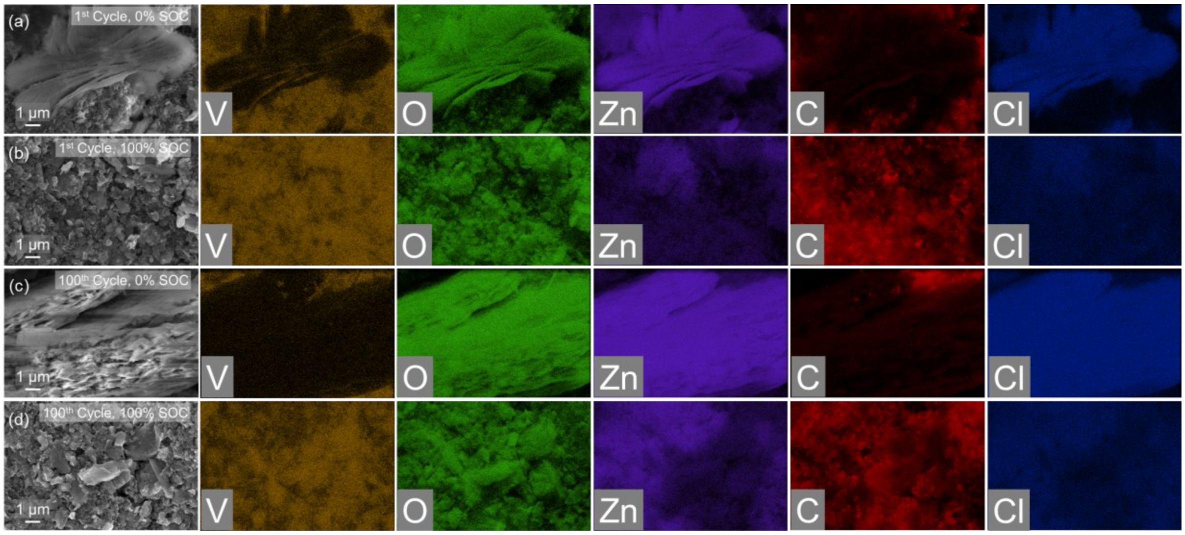

Figure S11. SEM images and EDS maps of V, O, Zn, C, and Cl taken for the MD-ZVO electrodes retrieved from AZIB cells containing the 2.6 M $Zn(OTf)_2$ electrolyte at (a) 1st cycle 0% SOC (Zn/V ratio = 1.284), (b) 1st cycle 100% SOC (Zn/V ratio = 0.138), (c) 100th cycle 0% SOC (Zn/V ratio = 11.571), and (d) 100th cycle 100% SOC (Zn/V ratio = 0.397). At 0% SOC, the surface becomes coated with ZCH flakes, which can be correlated with more intense O, Zn, and Cl maps. At 100% SOC, these flakes dissolve, leaving the electrode surface mostly pristine. Cl mapping of 100% SOC electrodes does show residual Cl that indicated some undissolved ZCH that may serve as seed crystals for ZCH formation during discharge. The elevated Zn/V ratios for electrodes retrieved from 100th cycle compared to 1st cycle also implies residual ZCH on electrode surface.

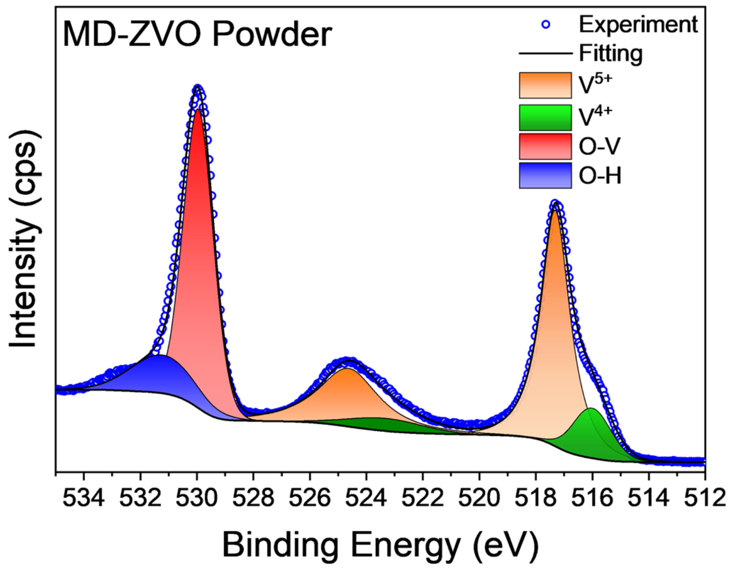

Figure S12. XPS spectrum of MD-ZVO powder in the V2p and O1s region with peak fitting.

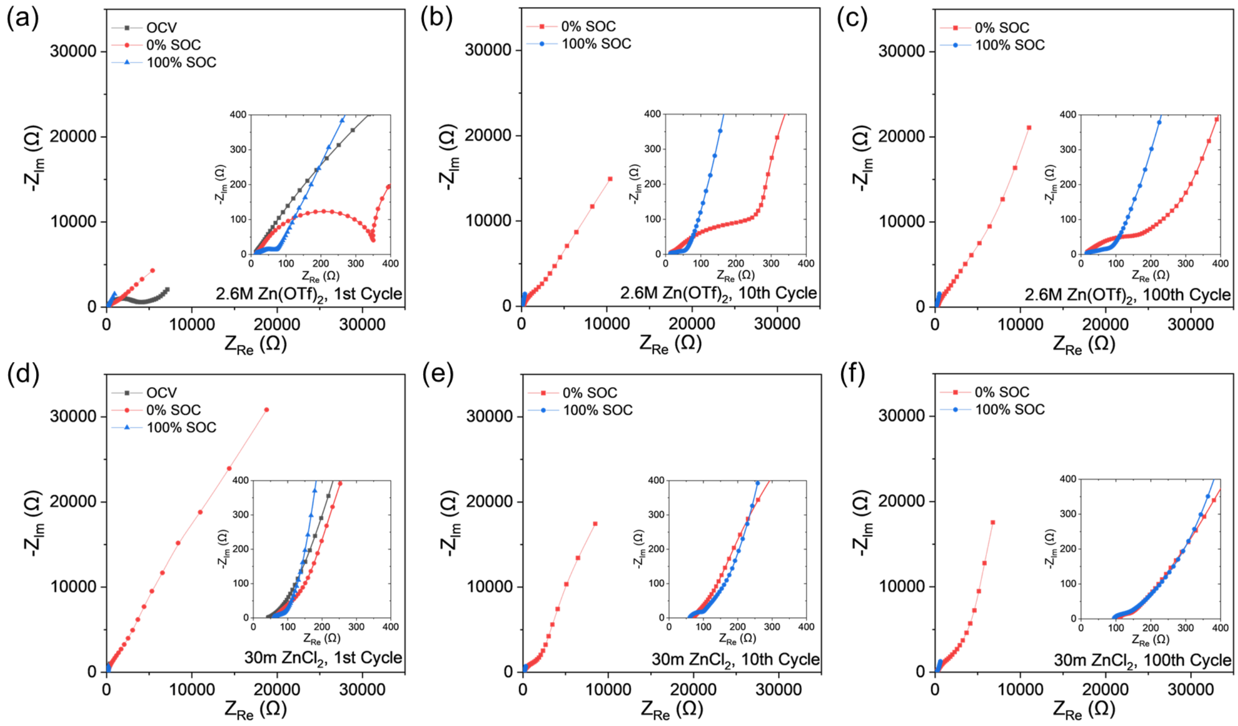

Figure S13. EIS spectra of aqueous Zn-ion cells containing MD-ZVO electrodes taken at (a,d) 1st, (b,e) 10th, and (c,f) 100th cycles using (a-c) 2.6 M $Zn(OTf)_2$ and (d-f) 30 m $ZnCl_2$ electrolytes. Insets show the subsection of the spectra in the high-frequency region.

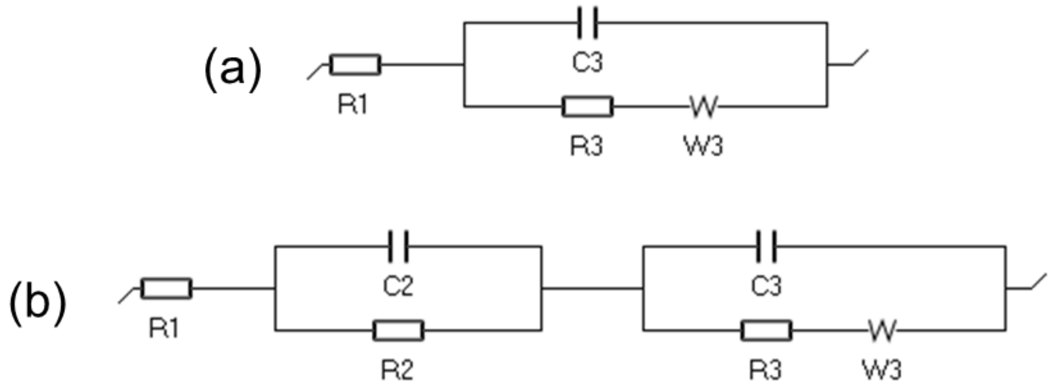

Figure S14. Equivalent circuit models used to fit the EIS spectra from **Figure S13**. (a) Randles circuit and (b) Randles circuit with additional surface layer.

Table S2. EIS fitting results for MD-ZVO cells with 2.6 M $Zn(OTf)_2$ and 30 m $ZnCl_2$ electrolytes.

**2.6 M $Zn(OTf)_2$**	R_1 (Ω)	C_2 (F)	R_2 (Ω)	C_3 (F)	R_3 (Ω)	s_3 (Ω $s^{-0.5}$)	X^2/Z^2	D (cm^2 s^{-1})
OCV	20.3			1.02E-06	2025	1197	12.55	1.62E-12
1st Cycle 0% SOC	15.39	5.69E-07	20.81	1.94E-06	217.9	679.2	1.425	5.03E-12
1st Cycle 100% SOC	16.1			3.30E-06	26.37	116.4	4.298	1.71E-10
10th Cycle 0% SOC	14.66	4.05E-07	15.51	3.51E-06	55.35	1096	4.091	1.93E-12
10th Cycle 100% SOC	15.93			1.41E-06	16.38	92.52	4.703	2.71E-10
100th Cycle 0% SOC	17.17	2.12E-07	19.49	1.02E-06	67.23	1229	4.543	1.54E-12
100th Cycle 100% SOC	19.74			1.42E-06	26.16	120.8	4.031	1.59E-10

**30 m $ZnCl_2$**	R_1 (Ω)	C_2 (F)	R_2 (Ω)	C_3 (F)	R_3 (Ω)	s_3 (Ω $s^{-0.5}$)	X^2/Z^2	D (cm^2 s^{-1})
OCV	43.08			6.88E-06	4.885	97.81	1.853	2.42E-10
1st Cycle 0% SOC	64.32	3.04E-05	80.16	2.02E-04	8.25E-05	4344	1.431	1.23E-13
1st Cycle 100% SOC	58.38			1.02E-05	10.98	69.16	2.128	4.85E-10
10th Cycle 0% SOC	68.83	3.01E-05	38.94	1.34E-04	1.96E-05	1613	1.521	8.91E-13
10th Cycle 100% SOC	61.42			5.56E-06	26.69	94.22	1.393	2.61E-10
100th Cycle 0% SOC	119.4	7.77E-06	68.64	9.25E-05	2.65E-05	1848	1.224	6.79E-13
100th Cycle 100% SOC	98.1			3.08E-06	32.52	176.5	0.999	7.45E-11

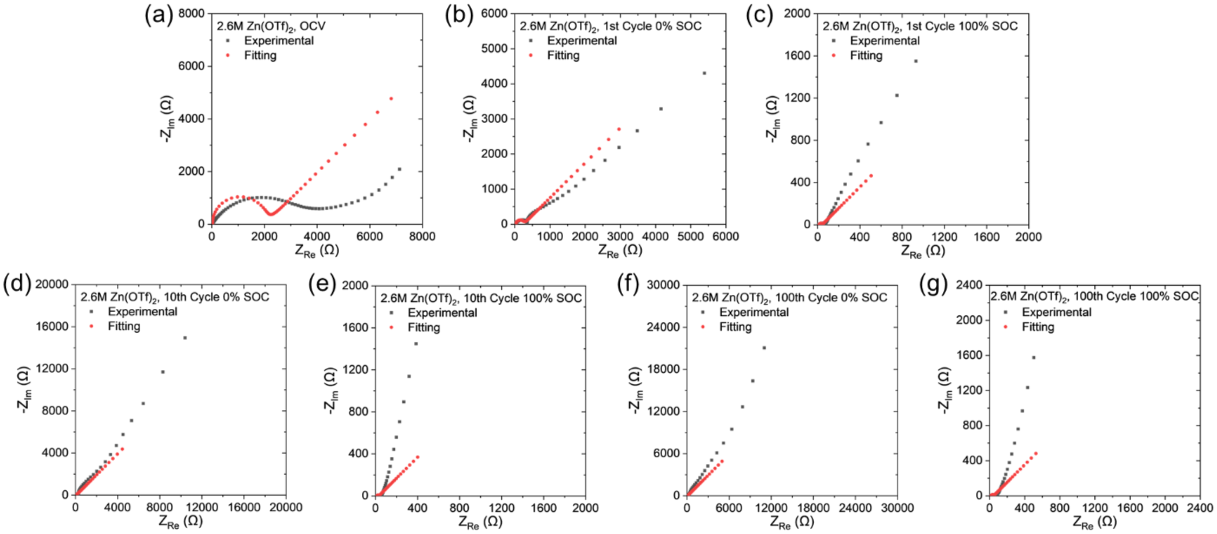

Figure S15. EIS experimental and fitted spectra from MD-ZVO cells containing 2.6 M $Zn(OTf)_2$ taken at (a) OCV, (b) 1st cycle 0% SOC, (c) 1st cycle 100% SOC, (d) 10th cycle 0% SOC, (e) 10th cycle 100% SOC, (f) 100th cycle 0% SOC, (g) 100th cycle 100% SOC.

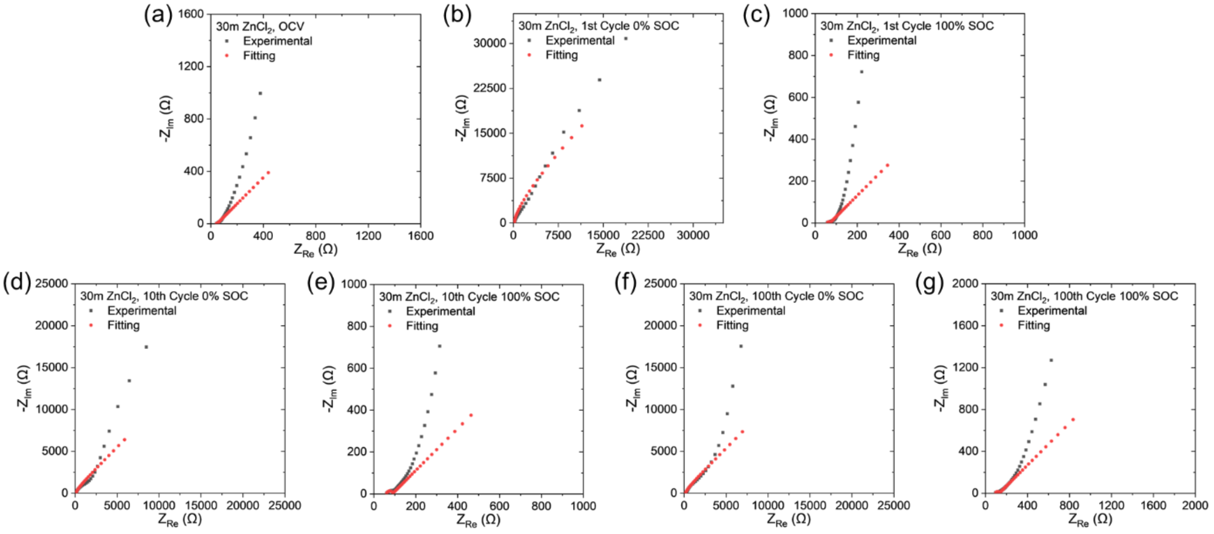

Figure S16. EIS experimental and fitted spectra from MD-ZVO cells containing 30 m $ZnCl_2$ electrolyte taken at (a) OCV, (b) 1st cycle 0% SOC, (c) 1st cycle 100% SOC, (d) 10th cycle 0% SOC, (e) 10th cycle 100% SOC, (f) 100th cycle 0% SOC, (g) 100th cycle 100% SOC.

Table S3. Comparison of performance of ZVO cathodes in AZIBs from literature.

Material	Electrolyte	Potential Window vs. Zn/Zn^{2+}	Initial Capacity @ Current Density	Ref
$Zn_{0.25}V_2O_5 \cdot nH_2O$	1M $ZnSO_4$	0.5 - 1.4 V	282 mAh g-1 @ 0.3 A g-1<br>260 mAh g-1 @ 2.4 A g-1	1
$Zn_{0.3}V_2O_5 \cdot 1.5H_2O$	3 M $Zn(OTf)_2$	0.3 - 1.6 V	426 mAh g-1 @ 0.2 A g-1<br>214 mAh g-1 @ 10 A g-1	2
$Zn_{0.1}V_2O_5 \cdot nH_2O$	2 M $ZnSO_4$	0.25 - 1.7 V	463 mAh g-1 @ 0.2 A g-1<br>240 mAh g-1 @ 10 A g-1	3
V_2CT_X-$Zn_xV_2O_5 \cdot nH_2O$	3 M $Zn(OTf)_2$	0.3 - 1.4 V	389.7 mAh g-1 @ 0.2 A g-1<br>223.9 mAh g-1 @ 10 A g-1	4
$Zn_{0.52}V_2O_{5-a} \cdot 1.8H_2O$	3 M $Zn(OTf)_2$	0.2 - 1.6 V	267.6 mAh g-1 @ 0.2 A g-1<br>165.1 mAh g-1 @ 20 A g-1	5
$Zn_mV_2O_5$	3 M $Zn(OTf)_2$	0.2 - 1.6 V	399.5 mAh g-1 @ 0.1 A g-1<br>90 mAh g-1 @ 20 A g-1	6
$Zn_{0.25}V_2O_5 \cdot H_2O$	1M $ZnSO_4$	0.5 - 1.4 V	300 mAh g-1 @ 0.06 A g-1<br>160 mAh g-1 @ 2.4 A g-1	7
$Zn_{0.162}V_2O_5 \cdot nH_2O$	3 M $Zn(OTf)_2$	0.2 - 1.6 V	355 mAh g-1 @ 0.05 A g-1<br>208 mAh g-1 @ 2 A g-1	8
$Zn_{0.99}V_5O_{12} \cdot 1.28H_2O$	3 M $Zn(OTf)_2$	0.2 - 1.6 V	401 mAh g-1 @ 0.2 A g-1<br>184 mAh g-1 @ 5 A g-1	9
**δ-$Zn_{0.19}V_2O_5 \cdot 0.57H_2O$**	**2.6 M $Zn(OTf)_2$**<br>**30 m $ZnCl_2$**	**0.2 - 1.6 V**	**450 mAh g-1 @ 0.1 A g-1**<br>**223 mAh g-1 @ 1.0 A g-1**<br>**315 mAh g-1 @ 0.1 A g-1**<br>**223 mAh g-1 @ 1.0 A g-1**	**This work**

Table S4. Comparison of performance of MXene-derived MVO cathodes in AZIBs from literature.

Material	Electrolyte	Potential Window vs. Zn/Zn^{2+}	Initial Capacity @ Current Density	Ref
V_2CT_x-$Zn_xV_2O_5·nH_2O$	3 M $Zn(OTf)_2$	0.3 - 1.4 V	389.7 mAh g-1 @ 0.2 A g-1<br>223.9 mAh g-1 @ 10 A g-1	4
$Mg_{0.2}V_2O_5·0.8H_2O$	3 M $Zn(OTf)_2$	0.2 - 1.6 V	346 mAh g-1 @ 0.1 A g-1<br>159 mAh g-1 @ 5 A g-1	10
$Mn_xV_{10}O_{24}·nH_2O@V_2CT_x$	3 M $Zn(OTf)_2$	0.2 - 1.6 V	358.2 mAh g-1 @ 1 A g-1<br>289.6 mAh g-1 @ 10 A g-1	11
$Zn_{0.99}V_5O_{12}·1.28H_2O$	3 M $Zn(OTf)_2$	0.2 - 1.6 V	401 mAh g-1 @ 0.2 A g-1<br>184 mAh g-1 @ 5 A g-1	9
**δ-$Zn_{0.19}V_2O_5·0.57H_2O$**	**2.6 M $Zn(OTf)_2$**<br>**30 m $ZnCl_2$**	**0.2 - 1.6 V**	**450 mAh g-1 @ 0.1 A g-1**<br>**223 mAh g-1 @ 1.0 A g-1**<br>**315 mAh g-1 @ 0.1 A g-1**<br>**223 mAh g-1 @ 1.0 A g-1**	**This work**

References

(1) Kundu, D.; Adams, B. D.; Duffort, V.; Vajargah, S. H.; Nazar, L. F. A high-capacity and long-life aqueous rechargeable zinc battery using a metal oxide intercalation cathode. *Nat. Energy* **2016**, *1* (10), 16119.

(2) Wang, L.; Huang, K.-W.; Chen, J.; Zheng, J. Ultralong cycle stability of aqueous zinc-ion batteries with zinc vanadium oxide cathodes. *Sci. Adv.* **2019**, *5* (10), eaax4279.

(3) Zhu, K.; Wu, T.; van den Bergh, W.; Stefik, M.; Huang, K. Reversible Molecular and Ionic Storage Mechanisms in High-Performance $Zn_{0.1}V_2O_5·nH_2O$ Xerogel Cathode for Aqueous Zn-Ion Batteries. *ACS Nano* **2021**, *15* (6), 10678-10688.

(4) Zhu, X.; Wang, W.; Cao, Z.; Gao, S.; Chee, M. O. L.; Zhang, X.; Dong, P.; Ajayan, P. M.; Ye, M.; Shen, J. Zn^{2+}-Intercalated $V_2O_5·nH_2O$ derived from V_2CT_x MXene for hyper-stable zinc-ion storage. *J. Mater. Chem. A* **2021**, *9* (33), 17994-18005.

(5) Liang, W.; Rao, D.; Chen, T.; Tang, R.; Li, J.; Jin, H. $Zn_{0.52}V_2O_{5-a}·1.8H_2O$ Cathode Stabilized by In Situ Phase Transformation for Aqueous Zinc-Ion Batteries with Ultra-Long Cyclability. *Angew. Chem. Int. Ed.* **2022**, *61* (35), e202207779.

(6) Zhang, S.; Chen, L.; Dong, D.; Kong, Y.; Zhang, J.; Liu, J.; Liu, Z. $A_mV_2O_5$ with Binary Phases as High-Performance Cathode Materials for Zinc-Ion Batteries: Effect of the Pre-Intercalated Cations A and Reversible Transformation of Coordination Polyhedra. *ACS Appl. Mater. Interfaces* **2022**, *14* (21), 24415-24424.

(7) Venkatesan, R.; Bauri, R.; Mayuranathan, K. K. Zinc Vanadium Oxide Nanobelts as High-Performance Cathodes for Rechargeable Zinc-Ion Batteries. *Energy Fuels* **2022**, *36* (14), 7854-7864.

(8) Liu, X.; Dong, X.; Passerini, S. Operando pH measurements revealing the promoted Zn^{2+} intercalation kinetics of pre-intercalated V_2O_5 cathode in aqueous zinc metal batteries. *J. Power Sources* **2024**, *623*, 235401.

(9) Xiao, W.; Yang, S.; Jiang, R.; Huang, Q.; Shi, X.; Tsang, Y. H.; Shao, L.; Sun, Z. V_4C_3 MXene-derived $Zn_{0.99}V_5O_{12}·nH_2O$ nanoribbons as advanced cathodes for ultra-long life aqueous zinc-ion batteries. *J. Mater. Chem. A* **2024**, *12* (9), 5530-5539.

(10) Guan, J.; Shao, L.; Yu, L.; Wang, S.; Shi, X.; Cai, J.; Sun, Z. Two-dimensional $Mg_{0.2}V_2O_5·nH_2O$ nanobelts derived from V_4C_3 MXenes for highly stable aqueous zinc ion batteries. *Chem. Eng. J.* **2022**, *443*, 136502.

(11) Zhu, X.; Cao, Z.; Li, X.-L.; Pei, L.; Jones, J.; Zhou, Y.-N.; Dong, P.; Wang, L.; Ye, M.; Shen, J. Ion-intercalation regulation of MXene-derived hydrated vanadates for high-rate and long-life Zn-Ion batteries. *Energy Storage Mater.* **2022**, *45*, 568-577.
